# Nuclear Mitochondrial DNA Activates Replication in *Saccharomyces cerevisiae*

**DOI:** 10.1371/journal.pone.0017235

**Published:** 2011-03-08

**Authors:** Laurent Chatre, Miria Ricchetti

**Affiliations:** 1 Departement d'Immunologie, Institut Pasteur, Paris, France; 2 Unité de Génétique Moléculaire des Levures, Institut Pasteur, Paris, France; University of Minnesota, United States of America

## Abstract

The nuclear genome of eukaryotes is colonized by DNA fragments of mitochondrial origin, called NUMTs. These insertions have been associated with a variety of germ-line diseases in humans. The significance of this uptake of potentially dangerous sequences into the nuclear genome is unclear. Here we provide functional evidence that sequences of mitochondrial origin promote nuclear DNA replication in *Saccharomyces cerevisiae*. We show that NUMTs are rich in key autonomously replicating sequence (ARS) consensus motifs, whose mutation results in the reduction or loss of DNA replication activity. Furthermore, 2D-gel analysis of the *mrc1* mutant exposed to hydroxyurea shows that several NUMTs function as late chromosomal origins. We also show that NUMTs located close to or within ARS provide key sequence elements for replication. Thus NUMTs can act as independent origins, when inserted in an appropriate genomic context or affect the efficiency of pre-existing origins. These findings show that migratory mitochondrial DNAs can impact on the replication of the nuclear region they are inserted in.

## Introduction

The transfer of genetic information from mitochondria, that are endosymbyosis-derived compartments, to the nucleus shaped the nuclear genome of eukaryotes [1]. In animals, only the transfer of DNA fragments (called NUMTs, for NUclear MiTochondrial) is active nowadays, while in plants ongoing transfer of entire genes has been reported [2]. As a consequence of this process, the nuclear genome of most eukaryotes including humans is colonized by NUMTs. The increasing number of eukaryote species analysed for their NUMT content allows these integrated sequences to be used as genetic tools to follow the geographic distribution of species or populations and to resolve phylogenetic ambiguities [3], [4].

We and others have previously shown that the insertions of NUMTs take place during the repair of DNA-double strand breaks in yeast [5], [6]. However, the mechanism(s) responsible for the transfer of genetic material from mitochondria to the nucleus as well as the factors that promote this process are unknown. Moreover, the role of NUMTs in nuclear chromosomes is unclear. Although originally considered as non-functional sequences, NUMTs can contribute to the evolution of nuclear genomes and occasionally participate in the formation of new exons [7] including functional exons in plants [8]. In humans, where, differently from yeast, they preferentially target genes and regulatory regions [7], NUMTs have also been associated with genetic diseases [9], [10], [11], [12]. The diversity of the pathologies due to NUMT insertions reflects a mutagenic process that can target a large spectrum of genes. Since NUMTs can potentially target any gene, and a continuous supply of DNA fragments of mitochondrial origin can be provided by the mitochondrial pool in the cell, new insertions of NUMTs may represent a threat in humans. The driving force underlying the insertion of these potentially harmful sequences is unknown. It has been speculated that NUMTs have a regulatory function as origins of replication [13], [14], promoters, or other genetic signals [6], [15]. Indeed, yeast mitochondrial replication origins activate replication of plasmids [13], [16]. Moreover, yeast mitochondrial DNA fragments of an average size of 1.2 kbp, and the complete mitochondrial genome of 5.5 kbp of a *petite* strain were shown to confer replication activity to plasmids [14], [17]. This finding led to the hypothesis that alternative DNA sequences could function as mitochondrial origins for *petite* strains, whose largely rearranged and size-reduced mitochondrial genome may lack classical replication origins. These data however did not address the origin activity of mitochondrial DNA in the nucleus. Moreover, they were not direct to mitochondrial DNA regions that have integrated the nuclear genome (NUMTs). It has also been postulated that NUMTs may activate oncogenes [15]. Accordingly, a NUMT insertion in an oncogene has been found in an established cell line [18] and the genome of rat cells of tumour origin appear to contain more NUMTs than the corresponding healthy cells [19]. In this context, we reported that two NUMTs with insertion polymorphism in humans are inserted in a tumour suppressor gene and in a potential angiogenesis factor, respectively, in apparently healthy individuals [7]. These elements suggest that NUMTs can be implicated in unregulated growth of somatic cells. However, no evidence for an active function of NUMTs in the nuclear genome has been reported. It has been recently found that a few NUMTs are inserted into promoter regions in the yeast *Schizosaccharomyces pombe*, where however they do not affect gene transcription levels on reporter plasmids [20]. In the same report, it has been described that most *S. pombe* NUMTs are located in regions that contain replication origins. Here we analyzed whether NUMTs can promote nuclear DNA replication in *Saccharomyces cerevisiae*.

## Methods

### Yeast strain and culture conditions

The *S. cerevisiae* strain FY69 (*MAT* ***a***, *leu2Δ1*), a S288C derivative, was used in this study. Yeast was grown at 30°C in either standard YPD medium or minimal medium lacking leucine (leu) to select for appropriate plasmids. Plasmid DNA (100 ng) was introduced into yeast using the standard electroporation method [21]. For 2D-gels were used also the *mrc1* (*MAT*a, *rad*5, *URA*3::*GDP-TK*, *mrc1*::*HIS*) and *rad53-11* (*MAT*a, *rad53-11*, *rad*5, *URA*3::*GDP-TK*) strains (a kind gift from P. Pasero).

### Plasmid constructs and mutagenesis

Vector pIRT2Δ originated by removal of the *Eco*RI*-Eco*RI ARS1 fragment (that contains the origin of replication of *Schizosaccharomyces pombe*) from the pIRT2 vector [22] (a kind gift from A. Holmes, Institut Pasteur). pIRT2 codes for the *LEU2* gene of *S. cerevisiae*. A variety of sequences were PCR amplified from yeast and plasmids using primers tagged with *Bam*HI sequences. Amplified sequences were then individually inserted, in both orientations, at the *Bam*HI unique site of pIRT2Δ. Each insertion was checked by digestion with appropriate restriction enzymes, PCR, and sequencing. Experiments with insertions only in the inverted orientation were performed with the vector pRS405 (insertions at the unique *BamHI* site), a YIP5 derivative [23], kindly provided by E. Fabre, Institut Pasteur; and with the vector pRS405-CEN that contains the yeast centromere CEN4 inserted at the unique *SalI* site of pRS405. In experiments with mutated ACS, the mutated sequence (5′ GCCGCGCGCCGCCGGCG) replaced the targeted ACS motif. A broad G+C rich substitution mutation for ARS elements was used in reference [24]. When more than one ACS motif was present, we mutated either the motif that had the highest identity to the consensus, or the one that was predicted to form the prevalent secondary structure according to MFOLD [25].

### Plasmid loss rate assay

A single yeast colony was used to inoculate 5-mL of YPD medium. For each construct, six independent yeast colonies were analyzed. Before culture growth, appropriate dilutions of the original inoculum were plated on selective (−leu) and nonselective (rich, YPD) media. The resulting colonies were counted and used to calculate the percentage of plasmid-carrying cells present in the initial culture (% I). The YPD culture was then incubated at 30°C for 12 generations during an 18 h period as in [26]. After incubation, appropriate dilutions were plated on selective and nonselective media. The resulting colonies were counted and used to calculate the percentage of plasmid-carrying cells present in the final culture (% F). The plasmid loss rate (PLR) was calculated according to the formula: PLR = 1−(% F/% I)^(1/n)^. The Mann-Whitney test, used to analyse the difference between the tested plasmid and the empty vector (or between wild-type and mutated sequences), was chosen due to the number of experiments (n = 6).

### Transformation efficiency

Yeast cells were transformed with a variety of plasmid constructs described above. The vector was pIRT2Δ, or pRS405, or pRS405-CEN. Transformation and analysis of the relative transformation frequency were performed as in reference [27]. The efficiency of transformation was evaluated by the number of transformant/µg of DNA, using 10^8^ competent cells/transformation. Transformation with the pRS405 empty vector resulted in no colony. For each construct, six independent experiments were performed.

### 2D-Gel Electrophoresis

Yeast were grown at 25°C in YPD medium until mid-log phase and arrested in G1 for 2.5 h with 2 µg/ml alpha-factor (GenePep, France). Cells were released from G1 by centrifugation and resuspension in fresh medium. When indicated, cells were resuspended in fresh medium containing 200 mM HU (Sigma) and exposed for 60 min. Total genomic DNA was extracted according to the protocol of the QIagen genomic DNA Handbook using genomic-tip 20/G columns. Neutral/neutral two-dimensional gel analyses were performed as described [28]. Replication intermediates were analysed by Southern blotting using specific probes against the fragment of interest. Probes specific for the examined fragments were created by PCR amplification of the region of interest. The reproducibility of the 2D-gel replication intermediates was tested for each sample.

### *In silico* analysis of ACS motifs

The OriDB (www.oridb.org) DNA Replication Origin Database was used as source and reference to collect the ACS expanded motifs, and localize ARS [29]. The SGD (http://www.yeastgenome.org) Saccharomyces Genome Database provided sequences and maps for comparative analysis. All the alignments were obtained *via* CLUSTALW DNA sequences from NPS@ server (http://npsa-pbil.ibcp.fr).

## Results

### NUMTs are rich in replication origin consensus motifs

**Figure 1:**
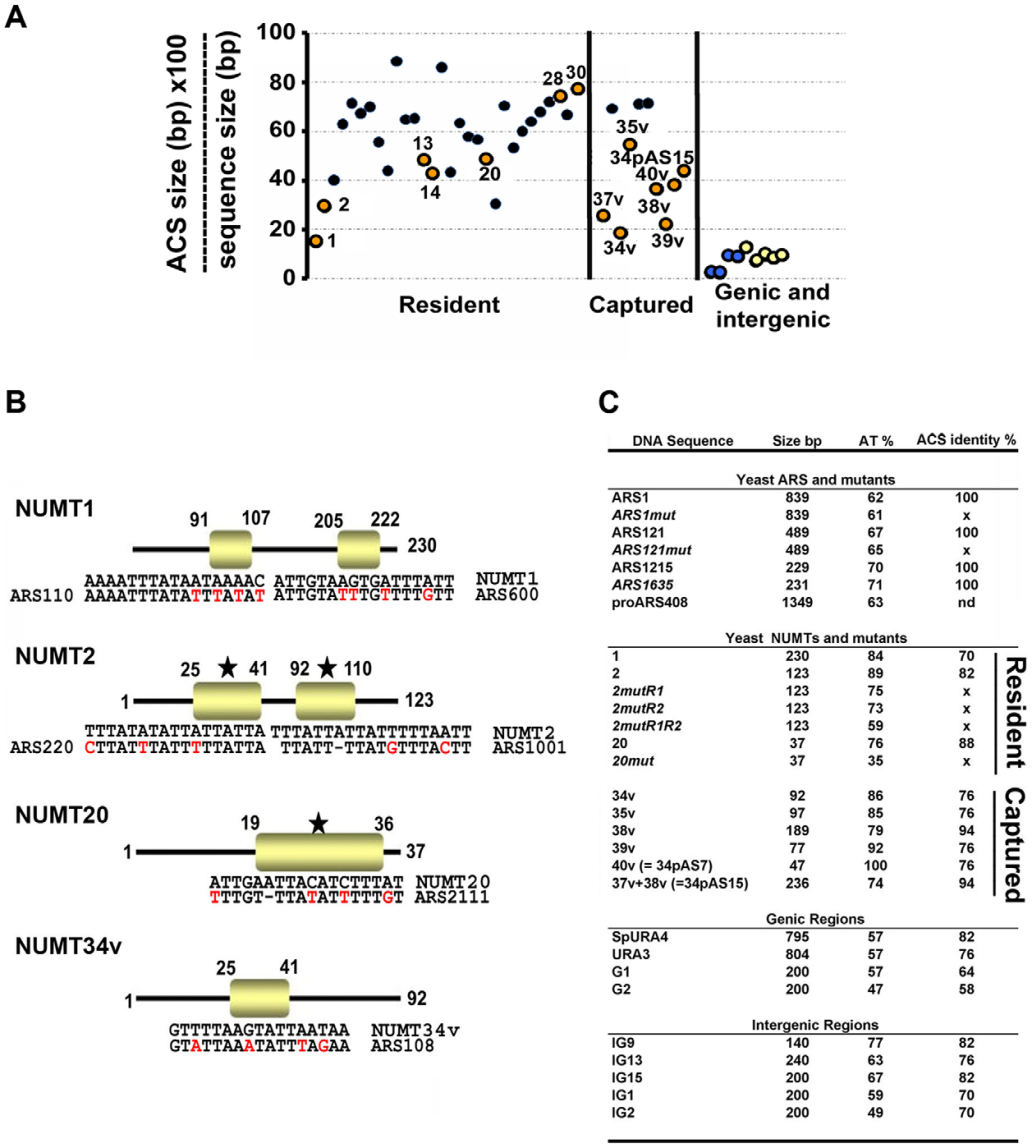
Paradigm to investigate origins of replication in *Saccharomyces cerevisiae*. (**A**) Density of ACS motifs within a given sequence. On the X-axis is indicated the type of sequence analysed, and on the Y-axis the percentage of the sequence (in bp) occupied by ACS motifs. Coloured dots indicate sequences that have been analysed in this study. In several cases the name of the sequence is indicated. Significantly different values for the group of *S. cerevisiae* NUMTs versus the group of genic and intergenic regions (p = 1.4×10^−11^) according to the t-Student test. (**B**) Sequence and position of the 17 bp ARS core-A motif (ACS) (rectangle) in several NUMTs. The sequence of confirmed ACS is shown below. A star indicates the ACS motif that has been mutated in this study. (**C**) Summary of the sequences, carrying either NUMT or non-NUMT sequences (Tables S1 and S2), inserted in pIRT2Δ, pRS405 and pRS405-CEN. Nd = not identified; Sp = *S. pombe*. “v” indicates captured NUMTs [5]. The nomenclature of yeast NUMTs is adapted from reference [5].

The replication origins of the budding yeast *S. cerevisiae*, also identified as ARS (autonomously replicating sequences), are ∼200 bp (or longer) and contain an essential 11-bp ARS core-A consensus sequence (ACS) [30], [31], extended to a 17-bp motif when based on a larger number of origins [32], [33]. Most origins contain multiple, imperfect matches to this motif with the best match not necessarily corresponding to the essential ACS. A match to the ACS is necessary but not sufficient for origin function [31]. We analyzed 31 NUMTs resident in the *S. cerevisiae* genome and 9 NUMTs captured in the yeast genome during the repair of DNA double-strand breaks (DSB) [5], (Table S1). We found that these NUMTs whose size ranges from 22 to 236 bp contained, with a few exceptions, one or more ACS motifs (70–94% identity to the 17 bp-ACS consensus, recalculated on 115 ACS sequences, Figure S1A). Accordingly, yeast NUMTs, but not control sequences including AT-rich elements, are distinctly ACS-rich (Figure 1A). With a few exceptions, more than 40% of the NUMT is occupied by ACS motifs, and this value rises to ≥70% for one third of NUMTs. Indeed, given the high density of ACS motifs, some NUMTs appear essentially as ACS “carriers”.

### High plasmid replication activity in the presence of NUMTs

**Figure 2:**
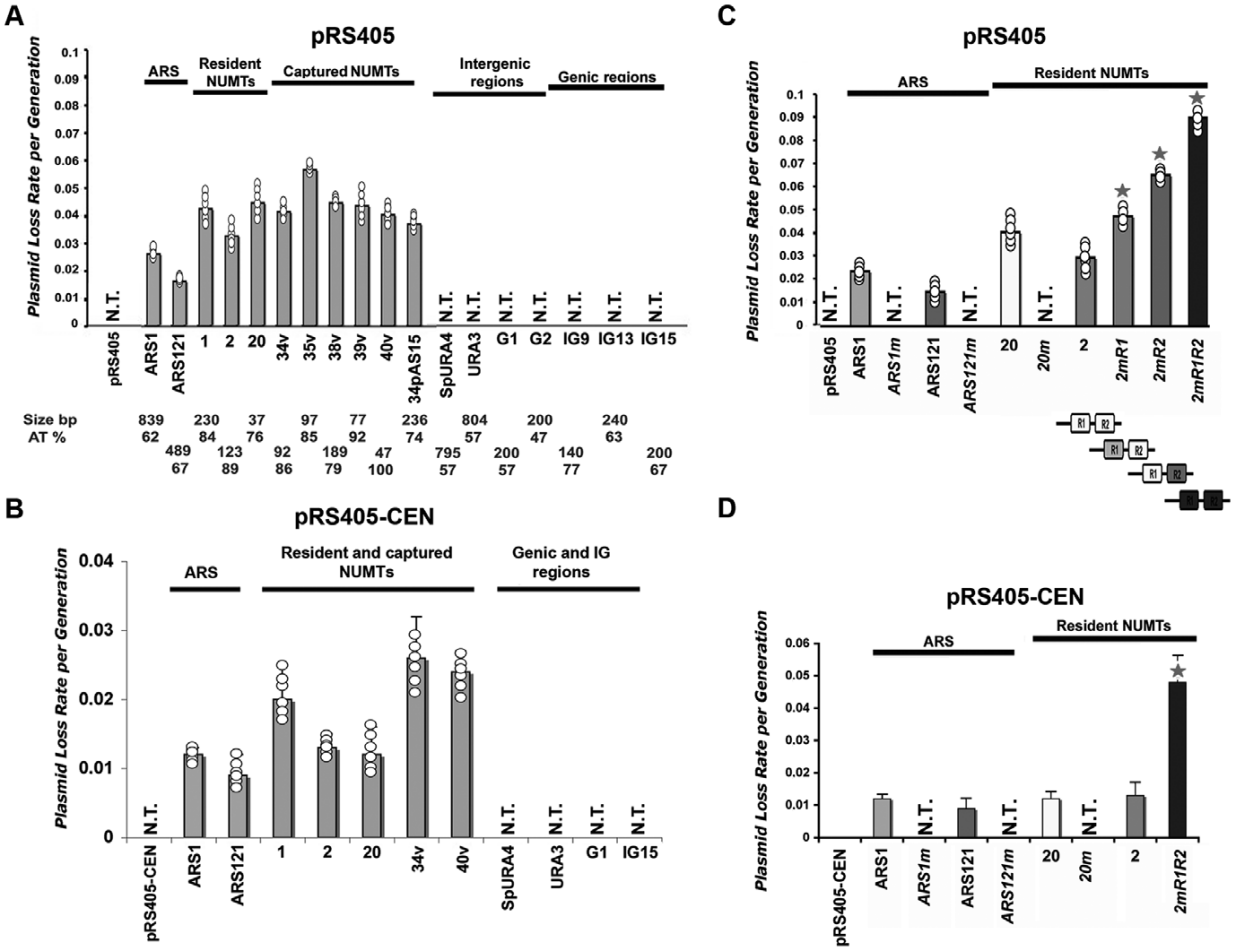
Plasmids carrying NUMTs display low loss rate in yeast. Plasmid loss rate per generation measured in 6 independent transformants (circles) per construct (average ± standard deviation). When values are identical, only one circle is shown. (**A**) Analysis with vector pRS405. *ARS1* and *ARS121* are positive controls. The empty vector as well as the insertion of random control sequences are unable to promote plasmid replication, therefore they do not support colony growth (N.T., for no transformants). Insertion of an ARS or a NUMT sequence activates plasmid replication and colony growth, therefore plasmid loss rate was measurable in these samples. The size and the AT content of each sequence are indicated in the bottom (**B**) Analysis with vector pRS405-CEN, a pRS405 derivative that contains the centromeric region CEN4. (**C–D**) Replication activity with mutated ACS. Plasmid loss rate following ARS and NUMT mutagenesis (“m”) to replace the 17-bp ACS motif in vectors pRS405 and pRS405-CEN. A star shows a significant difference between the mutated sequence and the corresponding original sequence (n = 6; p<0.05, Mann-Whitney test) (**C**) Vector pRS405. Mutation of the motif in both ARS and in NUMT20 results in complete loss of replication activity (no transformants, N.T). Mutation of one or both ACS motifs in NUMT2 (shown in dark rectangles in the scheme below) results in a progressive increase in plasmid loss rate. (**D**) Vector pRS405-CEN. Mutation of the motif in both ARS and in NUMT20 results in complete loss of replication activity (no transformants, N.T). Mutation of one or both ACS motifs in NUMT2 results in a progressive increase in plasmid loss rate.

We used plasmid maintenance assays in *S. cerevisae* [26], [33], to provide functional evidence that ACS-containing NUMTs act as origins of replication. The principle of the assay being that plasmids containing origins are maintained in extrachromosomal form in transformed yeast cells [30]. For this, we inserted individual yeast NUMTs, in one or both orientations in three types of plasmids. As control, we inserted well-characterized ARS1 [33] and ARS121, one of the most efficient known origins [27], [34], and a variety of other sequences (random genic and intergenic yeast sequences, see Figure 1C and Table S2). We then evaluated the plasmid loss rate per generation, revealing plasmids able to successfully replicate after subculturing in non-selective medium. Experiments were performed with six independent clones for each construct. We first checked whether NUMTs enhanced the replication activity of an ectopic vector, and used pIRT2Δ, a *Schizosaccharomyces pombe* vector that replicates very poorly in *S. cerevisae*. We found that NUMT and ARS, but not control random sequences, enhance dramatically the replication activity of pIRT2Δ (Figure S2A). We then checked whether NUMTs act as replicators *per se*, and inserted sequences of mitochondrial origins and controls sequences (in the inverted orientation) in vectors that have not detectable replication activity. pRS405 and pRS405-CEN that do not contain or contain a centromeric sequence, respectively, were used as vectors (the second type of vectors are more currently used in this type of studies [35]). Figure 2 shows that the results obtained with pIRT2Δ were exacerbated with pRS405 and pRS405-CEN, where plasmid loss rate was measurable only in plasmids containing an ARS (0.015–0.024 and 0.009–0.012, respectively) or a NUMT (0.030–0.052 and 0.012–0.026, respectively), while plasmids containing control sequences did not produce transformants. Importantly, NUMTs promote replication activity in spite of being remarkably shorter (37–236 bp) than ARS (489–839 bp). Thus, all tested NUMTs, as well as control ARS, confer replication activity to centromeric and non-centromeric plasmids, while random sequences do not.

There is no apparent correlation between plasmid loss rate and 1) size of the insert; 2) the level of identity to the ACS motif; or 3) the AT richness of the sequence (Figure S2B–D). Moreover, random control sequences rich in AT do not produce transformants (i.e. sequence IG9, 77% of AT-content, Figure 2A). Therefore we cannot ascribe our findings to random AT-richness of the sequences. In support of our conclusions, it has been shown that only 1–2% of a random AT-library of 80-mers (mono- and multimers) have some replication activity in yeast plasmids [36]. Conversely, random control sequences that also contain ACS motifs (identity to the consensus 64–82%) were unable to promote replication (no transformants produced when these sequences were inserted in vectors pRS405 and pRS405-CEN (Figure 2, see also Figure S2D). Taken together, these results show that NUMTs act as replication origins in plasmids and that the presence of ACS motifs *per se*, their level of identity to the consensus, and the size and AT content of the sequence is not sufficient to confer origin activity, as it is also the case for ACS in ARS [33].

### Mutation of the critical ACS motif reduces origin activity of NUMTs

To examine whether origin activity was conferred to NUMTs by the ACS motif, we constructed plasmids carrying ARS and NUMTs (in both orientations) where the 17-bp ACS motif was replaced with a G+C rich sequence. A broad G+C substitution mutation was previously described to test the functional importance of ARS elements [24]. When more than one ACS motif was present, we mutated either the motif that had the highest identity to the consensus, or the one that was predicted to form the prevalent secondary structure according to MFOLD [25]. Mutation of the (known) key ACS motif in ARS1 [37] and in ARS121 [33] in vectors pRS405 and pRS405-CEN did not gave rise to colonies, indicating that their replication properties were lost, as expected (Figure 2C–D). Importantly, this was also the case when mutating the single ACS motif in NUMT20, 37 bp in length. Reduction in replication activity (increased plasmid loss rate values) was observed after mutation of one ACS motif (R2), and of both motifs (R2+R1) in NUMT2; in the last case a few colonies did grow (pRS405 and pRS405-CEN), indicating that additional elements play a role in replication efficiency [26]. These results were confirmed with the ectopic vector pIRT2Δ (Figure S3). Thus, mutation of the critical ACS motif in NUMTs reduces the plasmid replication activity to background levels. However, as it is the case for ARS, the critical ACS motif cannot be predicted only by sequence analysis [31].

### High transformation efficiency in the presence of NUMTs

The replication activity of NUMTs was also demonstrated by the higher transformation efficiency of plasmids containing ARS and NUMTs, compared to plasmids carrying random sequences and mutated NUMTs in pRS405 and pRS405-CEN vectors (Table S3). Indeed, with vectors pRS405 and pRS405-CEN only plasmids containing ARS and NUMTs produced transformants (several hundreds and a few thousands of colonies/µg of DNA, respectively) while control sequences did not. Moreover, mutation of the ACS motif in colony producing NUMTs resulted in the complete loss (NUMT20) or in the decrease (NUMT2) of transformation efficiency, in agreement with plasmid loss rate data. With pIRT2Δ vector we observed a larger number of colonies when ARS and NUMTs were present, compared to the empty vector and to the presence of random sequences, with a reduction to background levels when the ACS motif was mutated (data not shown).

These results were also confirmed by dot colony assay (Text S1 and Figure S4). Taken together, three independent assays, and the usage of three different types of vectors show that all plasmids containing NUMTs, in either orientation, are indeed replicated in yeast while those including a variety of random yeast sequences, or mutated ARS and NUMTs are not.

### NUMTs as late replication origins in chromosomes

**Figure 3:**
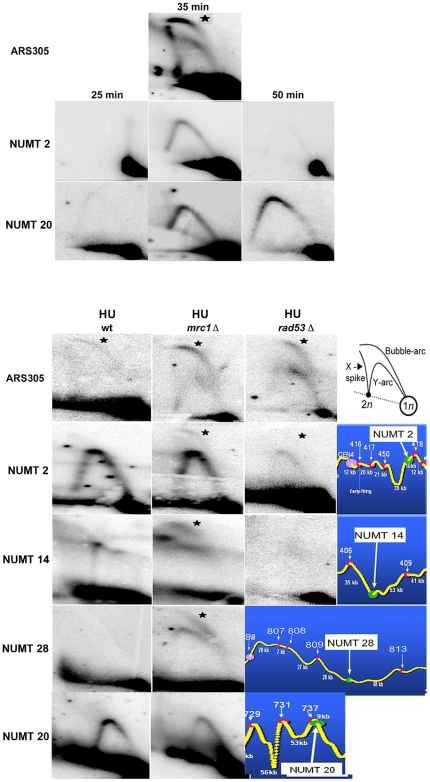
2D gels analysis of replication intermediates in chromosomes and localization of NUMTs at nuclear replication profiles. 2D-gel electrophoresis visualizes replication events at NUMTs in wild-type (wt), *mrc1* and *rad53-11* strains. Cells were arrested in G1 with alpha-factor and then released into YPD medium for 25 min, 35 min or 50 min alone (upper part of the figure), or in the presence of 0.2 M HU for 60 min (lower part of the figure). A scheme of the migration pattern in 2D-gel electrophoresis is shown. The extremely efficient ARS305 was used as control. Star indicates bubble molecules (origin firing). Probes specific for the examined fragments were created by PCR amplification of the region of interest. Fragments examined: ARS305, *EcoR*I 5.8 Kbp; NUMT2, *Hind*III 4.5 Kbp; NUMT14, *Eco*RI 7.4 Kbp; NUMT28, PstI 7.7 Kbp; FY1679, *Hind*III 5.4 Kbp; 34pBE28, *Hind*III 3.3 Kbp; 34pAS7, *Hind*III 3.35 Kbp; 34pAS15, *Hind*III 3.54 Kbp. A colour scheme (right, below) indicates the position of the NUMT according to the *S. cerevisiae* replication profiles reported in reference [42]. Yellow curve, replication time; peaks, origins of replication (early origins are higher); NUMTs, green circles; ARS, pink circles. ARS, named and indicated with a white arrow; NUMTs names, white rectangle. The distance between two ARS or between an ARS and a NUMT is indicated in kbp. CEN (a purple circle) corresponds to centromere; the Y-axe indicates “early” (top) and “late” (bottom) replication activity; trep, replication time in min. See Fig. S6 for the distribution of all NUMTs on replication profiles of the *S. cerevisiae* genome.

We then examined whether NUMTs function as origins of replication on the chromosome. To do this, restriction fragments centered on NUMTs were tested by two-dimensional (2D)-gel electrophoresis, and the presence of replication intermediates was detected with a radiolabeled probe [28]. A bubble signal is detected if DNA replication initiates within the central half of a restriction fragment. In contrast, passive replication from a neighboring origin generates a fork signal (Y arc). A time course analysis of replication intermediates centered on NUMTs 2 and 20 only revealed the presence of Y arcs in wild type cells at 35 minutes, indicating that these NUMTs are either inactive as chromosomal origins, or are passively replicated from adjacent origins before they have time to fire (Figure 3). To discriminate between these two possibilities, cells were treated with hydroxyurea (HU) in order to impede fork progression. Since late-firing origins are repressed by checkpoint pathways in HU [38], [39], we also used checkpoint mutants *rad53* and *mrc1* to analyze the replication profile of NUMTs. Importantly, NUMT2 as well as NUMT14 and NUMT28 show a weak but reproducible bubble arc in the presence of HU and in the *mrc1* mutant. This bubble arc was not detected in wt cells, indicating that these NUMTs act as late chromosomal origins. This was not the case for NUMT20 that does not show a replication bubble arc in the *mrc1* mutant, indicating that either it does not act as a replication origin or that its origin activity is too weak or it fires too late to be detected under these conditions. In *rad53* mutants, where replication forks collapse in the presence of HU [39] there is essentially no bubble signal for NUMT2 and NUMT14. Importantly, 2D-gels results on replication bubbles were further and fully confirmed in analysis of origins by BrdU incorporation and hybridization of BrdU-labeled DNA on Affymetrix tiling arrays on *mrc1* and *rad53-11* mutants, as well as on the *mec1-1* mutant that cause fork stalling and early activation of late origins, respectively [40], see Figure S5. This tiling arrays approach also evidenced other NUMTs that may function as independent origins, i.e. NUMT23, NUMT25, NUMT31 (Figure S5). Thus, the replication activity in these regions was also detected by independent assays that ignored the presence of a NUMT therein (see also below). In conclusion, at least three NUMTs appear to act as late replication origins at their location in the chromosome and this function is unprecedented for sequences of non-nuclear origin.

### About one half of NUMTs are located close to or within active chromosome origins

We then examined where NUMTs are located in chromosomes with respect to actual origins. Although about 350 ARS have been estimated in the genome of *S. cerevisiae*, only a subset of them appears to direct replication initiation in the S-phase [26], [41]. A previous investigation revealed the detailed topography (*i.e.* the location and the time of activation) of the most efficient origins in *S. cerevisiae* chromosomes based on oligonucleotide microarrays [42]. According to this data, most but not all ARS are located at replication peaks, while some ARS are located at valleys and others at intermediary positions. Indeed, several but not all ARS are active origins, according to replication profiles. Using this data, we positioned resident NUMTs on the replication profiles of yeast chromosomes (color panels in Figure 3 and Figure S6). With this analysis we found that about one third (10/28) of NUMTs are located on either early or late peaks corresponding to active origins (Table S4). Moreover, several NUMTs are located at peaks where no ARS is identified (orphan peaks), suggesting that the NUMT may be responsible for origin firing. Intriguingly, NUMTs 2 and 28 that we showed display late origin activity are located at orphan peaks whereas NUMT20 that does not, is located elsewhere. However, the level of confidence for peaks where NUMTs 2 and 28 are located is low (see last column on Table S4, data extracted from [42]). Although the position of NUMTs at previously identified replication peaks is intriguing, it may not be a direct indication of the firing activity, since NUMT14 that also displays late-firing activity, is located at a valley.

More powerful genome-wide replication profiles with mutants that reveal less efficient origins may further clarify the replication activity of NUMT-containing regions, as it was the case for Affimetrix tiling arrays (see above). In this context, we further analysed the position of NUMTs according to origins detected with a variety of other methods. In one case, we referred to genome-wide mapping of ORC and MCM2p binding sites on tiling assays [43] and considered stringent (ORC-MCM2 binding sites), intermediary (MCM2-only), and less stringent (ORC-only) ARS. We also examined data where ssDNA regions were detected as firing markers [44]. Finally we took in to account late origins defined into four classes: CDR 0, 1, 2 3 that show increasing dependence upon the Clb5p cyclin and an increasing confidence as origins [45]. A compilation of the position of NUMTs with respect to origins identified by these different approaches is shown on Table 1A and Table S5. These data confirm and extend our previous finding, revealing by one or more approaches that 22 out of 34 NUMTs are located in regions that activate replication. Importantly, these data also reveal that at least two NUMTs that we showed to act as late replication origins by 2D-gels are located at regions identified as origins by independent tests. Indeed, the short NUMT28 is at the centre of a 15 kbp region identified as origin by heavy-light microarray [42], that is also recognized as a CDR-1 late origin. No other origin sequences but NUMT28 have been shown to act in this region. A replication bubble at the level of NUMT28 was also identified by Affymetrix tiling arrays [40], as indicated above. NUMT 14 was also found located in a region of origin by at least two tests, and this NUMT has been investigated further due to its vicinity to a likely ARS (see below). Finally, the late replicating NUMT2 does not appear on a replication origin with the tests indicated above, although it was found to be at the centre of a replication bubble using Affymetrix tiling arrays on replication mutant strains [40]. In conclusion, most NUMTs are located in regions identified as origins of replication where they are either positioned far from ARS and may act as independent origin elements as it seems to be the case for NUMT2 and NUMT28, or are located close to ARS and may act as elements that strengthen or affect pre-existing origins.

**Table 1:** NUMTs and replication origins.

NUMT	Summary	Δ (bp) NUMT-ARS	ARS
**1**	**ORC-MCM2**	**0**	**Likely proARS902**
**1**	**ORC-MCM2**	**216**	**ARS1002**
2	-	10426	ARS418
3	-	0	*Dubious ARS*
4	-	1391	*Dubious ARS*
**5**	**MCM2/ ssDNA/ CDR-1**	**0**	**Likely ARS**
**6**	**CDR-1**	**3400**	***Dubious ARS***
**6**	**CDR-1**	**15908**	**ARS 717**
**7**	**ORC-MCM2/ ssDNA**	**366**	**ARS 1014**
8	-	2256	Likely ARS
**9**	**CDR-1**	**15957**	**ARS 717**
**9**	**CDR-1**	**3320**	***Dubious ARS***
**10**	**ssDNA**	**292**	**ARS 1308**
**11**	**ssDNA**	**193**	**ARS 1308**
**12**	**ORC-MCM2/ ssDNA**	**114**	**ARS 923**
**13**	**ORC-MCM2/ ssDNA**	**0**	**ARS 1215**
**14**	**ssDNA / CDR-3**	**387**	**Likely proARS408**
**15**	**CDR-1**	**16045**	**ARS 717**
16	-	1490	*Dubious ARS*
**17**	**MCM2/ CDR-1**	**580**	**ARS 721**
18	-	0	*Dubious ARS*
**19**	**CDR-1**	**3284**	***Dubious ARS***
20	-	8627	ARS 737
**21**	**MCM2/ ssDNA**	**625**	**ARS 316**
**22**	**ssDNA**	**1440**	**Likely ARS**
23	-	13879	*Dubious ARS*
24	-	1412	*Dubious ARS*
25	-	1768	Likely proARS1632
26	-	11389	ARS 1113
**27**	**CDR-2**	**0**	***Dubious ARS***
**28**	**CDR-1**	**0**	***Dubious ARS***
29	-	28750	ARS 718
**30**	**ORC-MCM2/ ssDNA/ CDR-1**	**0**	**ARS 1635**
**31**	**ssDNA/ CDR-2**	**2922**	**Likely proARS1202**

Summary of the presence of NUMT at replication origins. This table summarize data presented in Table S5. The first column indicates the NUMT, and the second column the presence of the NUMT at replication origin according to one or more tests (the type of test is indicated). The third column indicates the distence (in bp) between the NUMT and the closest ARS element. The last column indicates the type of ARS, according to OriDB:“ confirmed” are origins that have been detected by ARS assay or 2D-gels, “likely” ARS are probable origins identified by two or more microarray studies and “dubious” are sites proposed to be origin only by one microarray-based study. Lines in bold (22/34) indicate NUMTs located at replication origins. Since multiple insertions occurred at some *loci*, the number of loci with NUMT(s) is 28, while individual NUMTs are 34.

### NUMTs overlapping with ARS are essential for replication

To check whether NUMTs impact on the replication activity of neighbour ARS, we first identified NUMTs located close to ARS in chromosomes, using the OriDB database [29]. This database includes confirmed, likely, and dubious ARS, defined according to the number of independent test(s) that show an origin at a give site. We found that at least 26% (9/34) of NUMTs are located close (<650 bp) to confirmed ARS, and this percentage increases to 47% (16/34) if we also take into account all ARS, including likely and dubious ARS (Table 2). The localisation of NUMTs close to ARS does not appear to be coincidental (p<0.001, see Text S1). The remaining NUMTs were located at a larger distance (in 10/34 cases between 651 and 5 kbp, and in 8 /34 cases at a distance >5 kbp) from ARS. Thus, almost half NUMTs are located close to ARS.

**Table 2:** NUMT-ARS distance.

Distance NUMT-ARS (bp)	Nb of NUMTs	%
0	8	23.5
1–650	8	23.5
651–5000	10	29.5
>5000	8	23.5

The table indicates the number of NUMTs, and the relative percentages, for each group of distances (bp), according to values shown in Table 1.

Among 16 NUMTs located close to ARS, eight are actually located within ARS. Two of these NUMTs (NUMT13 and NUMT30) are located within confirmed ARS, and six more NUMTs within likely and dubious ARS, whose coordinates to date include a rather large region. It is unclear whether NUMTs located within likely or dubious ARS function as key elements that promote replication, as it seems to be the case for NUMT28 analysed also by 2D-gel (see above, Figure 3) and Affymetrix tiling arrays [40], or whether replication is promoted by ARS sequences present nearby and which have not yet been identified. On the contrary, in cases where NUMTs and ARS are distinctly identified, we investigated in more detail the contribution of the various sequence elements in promoting replication (see below).

**Figure 4:**
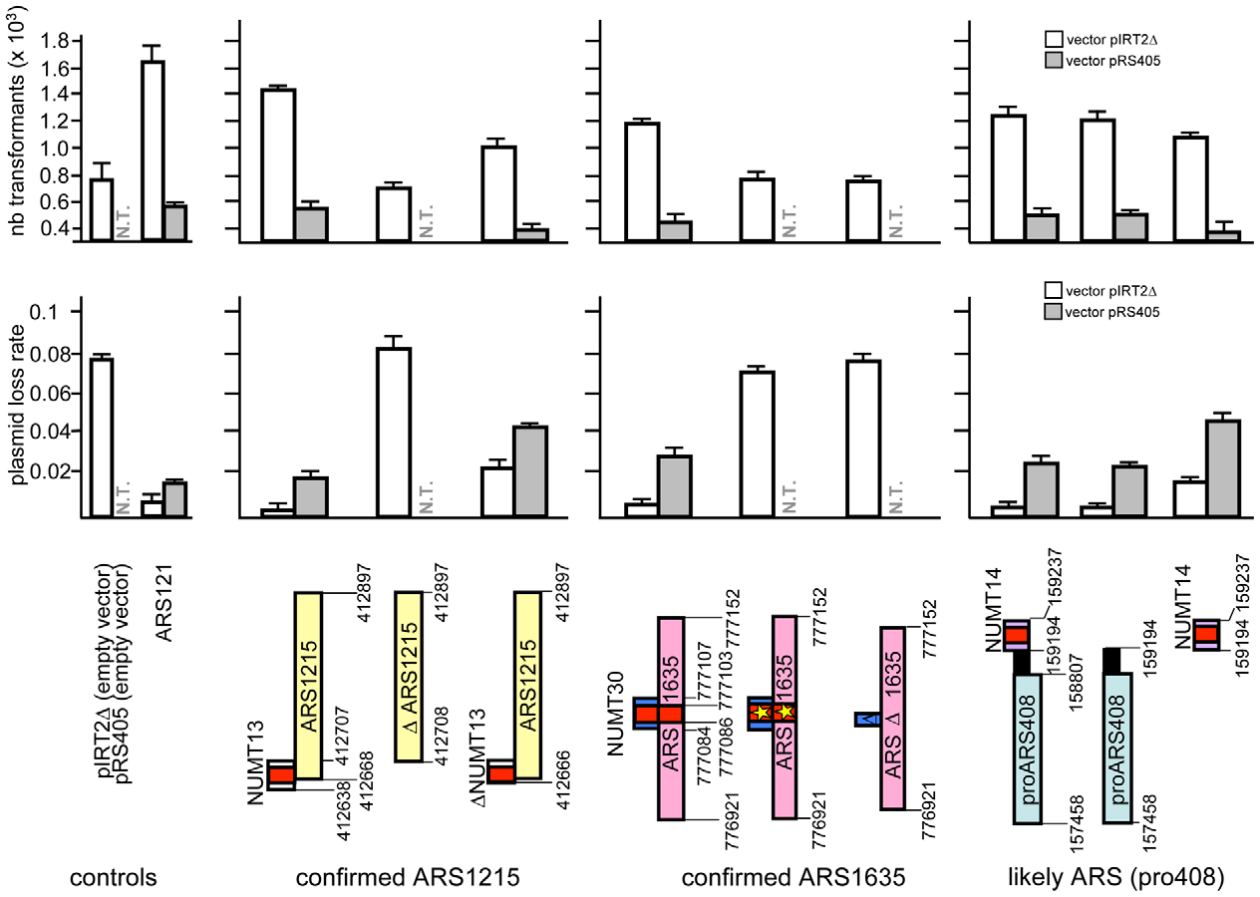
Replication efficiency of NUMT in ARS containing regions. Transformation efficiency (upper panels) and plasmid loss rate (middle panels) of sequences containing different combinations of an ARS and a NUMT, full sequences or various portions of them (schematized in the lower panels). NUMT13 (69 bp) overlaps by 39 bp the 5′ portion of ARS1215 (229 bp). NUMT30 (23 bp) is located in the middle of ARS1635 (231). NUMT14 (43 bp) is distant 387 bp from proARS408 (1349 bp); the definition of proARS is according to OriDB, since its activity has not been experimentally confirmed. For each of the three NUMT/ARS combinations, the full sequence and various portions of it (schematized in the lower panels) were analysed. For each construction, vectors pIRT2Δ (white bars) and pRS405 (grey bars) were used. The 17 bp ACS motif of NUMTs and ARS is indicated in red; no confirmed or predicted ACS element is reported for proARS408. A star indicates that the ACS motif has been replaced with a G+C rich region. The chromosomal coordinates of NUMTs and ARS are indicated; when portions of the full sequence are analysed, their coordinates are also indicated.

To assess the contribution of NUMTs in ARS containing regions, we measured the replication efficiency of vectors containing both a NUMT and an ARS, or a deleted/mutated portion of them. We tested three ARS-NUMT combinations: i) the confirmed ARS1215 that partially overlaps NUMT13 in its 5′ region, ii) the confirmed ARS1635, that fully overlaps NUMT30, and iii) proARS408, a likely ARS located 387 bp away from NUMT14 (Figure 4, lower panels). Plasmid loss rate assay and transformation efficiency were performed each on pIRT2Δ and on pRS405 vectors. Figure 4 shows that complete deletion (69 bp) of NUMT13 (which also deletes 39 out of 229 bp of ARS1215) results in plasmid loss rate and transformation efficiencies comparable to those of empty vectors. Deletion of just 28 bp of NUMT13 (which leaves an intact ARS1215) results in increased plasmid loss rate and reduced transformation efficiencies, indicating that this short region of NUMT13 strongly contributes to the replication efficiency of the region. Therefore, NUMT13 is essential to the replication activity of the ARS1215 containing region. This is also the case for NUMT30 (that fully overlaps ARS1635) whose partial deletion or mutation is sufficient to reduce the efficiency of replication to the levels of empty vectors. These results show that the NUMT component of ARS1635 and ARS1215, and in particular the ACS motif, is essential for replication activity since its disruption is associated with the loss of replication activity of the entire region. Finally, in a different NUMT-ARS arrangement, non-overlapping NUMT14 and proARS408 both have independent replication activity although proARS408 alone has replication activity levels comparable to those of the two elements together, at least on a plasmid assay. Importantly, we showed above that NUMT14 acts as a late replication origin in its chromosomal location, and the structure of the DNA fragment analysed herein shows that the bubble arc is centred on the NUMT and not on the neighbour ARS (see Figure S7). It is thus possible that in the chromosomal context the replication activities of close ARS and NUMTs are additive. Taken together, these data, resulting from two different assays and two independent vectors, indicate that NUMTs that partially overlap ARS or are located within ARS are essential for origin activity of the region. Thus NUMTs, non only can act as independent origins, but can also contribute to the origin activity of other sequences.

## Discussion

NUMTs are integrated during the repair of DNA breaks in yeast [5], [6]. The possible function of DNA sequences of mitochondrial origin in the nuclear genome has not been elucidated. We show that NUMTs have structural and functional properties of replication origins in yeast. By functional assays based on plasmid maintenance we show that all tested NUMTs, either resident in the genome or captured during the repair of DNA double-strand breaks, confer replication activity comparable to that of known ARS, including the highly efficient ARS121. Analysis with three distinct vectors, characterized by different replication and segregation properties, all lead to the conclusions that NUMTs confer replication activity, as control ARS do. Our data also underscore the role of key ACS motifs in the replication properties of NUMTs. There appear to be no differences in the way NUMTs and ARS promote replication of these vectors, in spite of NUMTs being much shorter than ARS (37–236 bp and 489–839, respectively, for the sequences tested here). Indeed, as it is the case for ARS, all NUMTs are active in both orientations and mutation of the key ACS motif reduces the replication activity to that of empty vectors. In our experiments a G+C rich mutation, used as in a previous study to test the functionality of ARS [24], replaced the original ACS motif. Although the high percentage of G+C in this sequence may involve a change in the helical stability of the DNA, we observed that the replacement of certain motifs was largely ineffective, indicating that the sequence itself is not responsible for the loss of replication activity. Again as in ARS, in NUMTs the key ACS motif cannot be identified just by its level of identity to the consensus sequence or its putative secondary structure stressing the concept that still unidentified sequence parameters contribute to the origin activity and to its efficiency. Furthermore, like ARS [42], almost all NUMTs are located in intergenic regions [5].

Thus, the ACS-dependent replication activity of ARS and NUMTs is not comparable with the occasional replication of yeast plasmids promoted by some non-yeast sequences. These few sequences resulted from the screening of thousands of megabases in genomes of many diverse organisms (for example see [46], [47], [48]), an occurrence statistically equivalent to chance, and were not shown to function in chromosomes. Our study provides direct evidence that a distinct family of natural non-nuclear yeast sequences, generated from a single 85.8 kbp molecule, confers DNA replication activity.

One of the most intriguing characteristics of NUMTs is their richness in ACS motifs, which may function as key replication sequences. The shortest NUMTs contain essentially the ACS motif(s) and longer NUMTs carry several ACS motifs, generally covering 40–80% of the entire sequence. This is the case for resident and for captured NUMTs. Thus, some NUMTs appear essentially as ACS “carriers”. This is not the case for random sequences and even for the mitochondrial genome itself, whose concentration of ACS motifs is remarkably low (about 1/44 of the total mitochondrial genome, see Text S1). Hence, mitochondrial DNA fragments that have successfully integrated the nuclear genome correspond to sequences that are essentially ACS-rich. NUMTs lacking ACS sequences might have also integrated into nuclear genomes. If this were the case, they have drifted sufficiently for them not to be recognised as NUMTs. However, the recent capture of NUMTs which are essentially rich in ACS-motifs (Figure 1A) at induced DNA breaks [5], supports the notion that successful integrations consist of ACS-rich NUMTs. Thus, NUMTs appear as special regions within the entire mitochondrial genome, characterized by ACS-richness. In this context, we checked whether the mitochondrial genome of *S. cerevisiae* contains more ACS motifs than those already transferred to the nuclear genome during evolution or captured at DNA breaks, and we found at least 43 additional motifs (Table S6). Given the role of the ACS motifs in the replication activity of NUMTs, we predict that new NUMT insertions in yeast chromosomes should contain one of the previously detected [5] or one of these newly identified motifs.

NUMTs function as replication origins not only in plasmids, but likely also in chromosomes. This replication activity on chromosomes is unprecedented for sequences of non-nuclear origin. We showed by 2D-gels that residents NUMTs, stably integrated in the nuclear genome, appear to function as late replication origins. Global chromosome analyses like 2D-gels usually reveal the most efficient origins [41], [42]. Therefore, mutants that determine adverse conditions for replication initiation or factors that limit the most efficient origins are used to identify other origins or determine the conditions and the hierarchy of usage [26], [44]). This was the case for NUMT2, NUMT14, NUMT28, that were revealed as late-firing origins after slowing down the replication of early origins nearby by treatment with HU in the *mrc1* checkpoint mutant. The origin function of these NUMTs was further confirmed by analysis of origins by BrdU incorporation and hybridization of BrdU-labeled DNA on Affymetrix tiling arrays in one or more mutants affected on fork progression (*mrc1*, *mec1-1*, *rad53-11*, [40]). This tiling arrays approach also evidenced other NUMTs that may function as independent origins. Furthermore, at least NUMT14 and NUMT28 were found to be located at origins identified with a variety of genome-wide tests that ignored the presence of the mitochondrial sequence therein [42], [44], [45]. For example, NUMT28 is located at the center of a large region identified as origin, in the absence of other replication elements, and it is very likely that the mitochondrial sequence promotes the firing of this region, in agreement with our 2D-gel results. However, since the origin activity of this chromosomal region was not checked in the absence of NUMT28, we cannot exclude that the firing activity is promoted by a nearby ARS, however, this is at present undetected. All together, these findings strongly suggest that at least some NUMTs function in the nuclear replication program as independent elements.

To date, the role of different components in the replication of the yeast genome and the impact of origins of different strength have not been completely unfolded. Origins appear to range in efficiency from those that are active in almost every cell cycle to those that are used in a small proportion of cell cycles. Origins can be less efficient because they are not competent to fire in some cells, because they are replicated passively in other cells in spite of being competent to fire, or both. The discovery that NUMTs impact on nuclear replication adds a further element to understand the role played by different sequences in the regulation of the DNA replication program. NUMTs may act as individual origins when inserted in an appropriate genomic context, as it is the case for late-firing origins shown above. The insertion in a less favorable genomic context may explain why some NUMTs (like NUMT20) where not found to function as chromosomal replication origins, at least under the tested conditions. NUMTs may also increase the efficiency of pre-existing origins, as it could be the case for NUMTs located close to or within ARS. In support of this hypothesis, we showed that two ARS-overlapping NUMTs, provide the essential ACS motif for replication activity on plasmids. This aspect is intriguing since a significantly large percentage of NUMTs are located close to ARS thus, possibly affecting the replication efficiency of these origins. Consequently, new NUMT insertions may modulate the efficiency of origins in the regions that they colonize. In this context, we previously reported the insertion of NUMTs at induced DSBs [5] at only two out of ten non-coding regions of the chromosome XI analysed [49], leading to the hypothesis that insertion of NUMTs may have also occurred at other loci but these insertions were selected out because they interfered with the local replication schedule.

NUMTs may contribute to replication *via* their original sequence or its successive mutation. In this context, we checked mutations in resident NUMTs, compared to the corresponding mitochondrial sequence (captured NUMTs, that are recent insertions, are 100% identical to the mitochondrial DNA). We found that both ACS and non-ACS motifs of NUMTs globally undergo pyrimidine enrichment, and increase in the GC content (Figure S8). We also observed that NUMTs that promote replication in plasmids are either identical, or their ACS motifs are, to the corresponding mitochondrial sequence (NUMTs 1, 2, and 20), or carry a single mutation in the ACS motif (NUMTs 13 and 30). We also noticed that the other NUMTs active in late chromosomal replication (NUMTs 14, 28) carry 1–2 mutations in ACS motifs that either decrease or increase the ACS consensus, respectively (not shown). Thus, by sequence analysis, it appears that non-mutated NUMTs are functional to promote replication while it is not clear, at least with the present set of data, whether point mutations in the ACS motifs or in the entire NUMTs affect the replication properties of these sequences.

It is also possible that the presence of NUMTs close to ARS is due to preferential insertion of mitochondrial sequences at origin regions, as it seems to be the case for *S. pombe* [20]. Indeed in the fission yeast frequent DSBs arise in origin regions under conditions of excessive firing. This finding supports the hypothesis of DSB-driven insertions to explain the almost exclusive presence of NUMTs close to ARS in *S. pombe*. However, NUMTs have not been found to act as independent origins in *S. pombe*, at least with chromatin immunoprecipitation experiments of origin recognition complex protein1 (Orc1) [20]. Moreover, deletion of a NUMT located close to an origin in *S. pombe* did not affect the binding of Orc1 to the origin site, and presumably its activity [20]. It is possible that NUMTs act differently in *S. cerevisae* and in *S. pombe*. In this context, differently from *S. cerevisiae* where half NUMTs are located far from ARS, in *S. pombe* the large majority (11/12) of NUMTs are located close to ARS. Moreover, in *S. cerevisiae* several NUMTs are located within regions defined as origins, including confirmed origins, while this does not appear to be the case in *S. pombe*. However, in case new ARS sequences will be identified in these organisms, the proportions reported here may change. Furthermore the sequence requirements of origins may be rather different in the two organisms. Alternatively, it is possible that the origin activity of NUMTs has not been detected in *S. pombe* because the analysis revealed only the most efficient origins, and NUMTs may function as late or weak origins. This was indeed the case for *S. cerevisae*, where NUMTs were detected as origins using conditions that slow down replication of the strongest origins. Finally, as it is the case for *S. cerevisiae* where some NUMTs promote replication, while others do not appear to do, it is possible that the NUMTs tested in *S. pombe* do not affect replication. As it was mentioned by Lenglez et al [20], it is not impossible that longer NUMTs may create new origins in *S. pombe*, as it was the case in a extrachromosomal context.

In the case of *S. cerevisiae*, we showed that at least some NUMTs act as independent origins, and that other NUMTs provide key elements for the replication. The potential significance of the origin activity of NUMTs is underscored by the fact that the insertion of NUMTs is an ongoing process [5], [7] and that their integration in the nuclear genome is not likely to be ceased in eukaryotes. It is tempting to hypothesize that NUMTs act as additional regulators of the cellular replication program in other eukaryotes. If a correlation between NUMTs and replication activity exists in other eukaryotes, it may explain the highly variable number of NUMTs fixed in the genome of different organisms [2], [50], which would be linked to the DNA replication architecture of each genome. Alternatively, the colonization and maintenance of NUMTs in the nuclear genome may be linked to other functions of these sequences or be due to other causes, like the occurrence of DSBs. Finally, these findings provide a new perspective on understanding the presence of mitochondrial DNA sequences in the nuclear genome of eukaryotes.

## Supporting Information

Figure S1 **ARS Core-A consensus sequences (ACS).** (**A**) Current 11 bp ARS core-A consensus sequence according to references [30], [51] (top). LOGO representation of the 17 bp-ARS core-A consensus sequence calculated on a set of 115 ARS available in the OriDB database and used in this study (middle). LOGO representation of the 17 bp consensus sequence calculated on a set of 43 mitochondrial ACS motifs (below). Square represents the 11-bp consensus. LOGO representation is designed by WebLogo3: Public Beta [52], [53]. (**B**) The size of each *S. cerevisiae* NUMT is plotted against the AT content. Circled NUMTs were investigated in this paper.
(TIF)

Figure S2 **Plasmid loss rates and sequence features analysis of the ectopic vector pIRT2Δ carrying a variety of sequences.** Plasmid loss rate per generation measured in 6 independent transformants (circles) per construct (average ± standard deviation). (**A**) Analysis with vector pIRT2Δ. For each sequence, the insertion was analyzed in both orientations (forward and reverse arrow). When values are identical, only one circle is shown. A black star indicates a difference versus empty pIRT2Δ, according to Mann-Whitney test (p<0.05). pIRT2Δ (no known ARS) and pIRT2 (containing an origin of replication of *S. pombe*, ARS1 that is expected to be inactive in *S. cerevisiae*) represent negative controls. *ARS1* and *ARS121* are positive controls. Plasmid loss rate plotted against (**B**) the AT content (%), (**C**) the sequence size, and (**D**) the identity to the 17 bp-ACS consensus. Framed orange triangles represent *S. cerevisiae* NUMTs. ARS are indicated with framed purple squares and other sequences with framed circles (yellow for intergenic and blue for genic sequences). Mutated sequences (“m”) are represented by empty triangles (NUMTs) or empty squares (ARS). Original NUMTs and ARS show low plasmid loss rate (high replication) independently from the parameter analysed on the X-axis. Data refer to experiments performed with pIRT2Δ.
(TIF)

Figure S3 **Replication activity with mutated ACS.** Plasmid loss rate following ARS and NUMT mutagenesis (“m”) to replace the 17-bp ACS motif in vectors pIRT2Δ. (**A**) Mutated sequences are represented by grey towers; different intensities of grey are used for mutation of more than one motif. ARS are mutated in the ARS core-A motif [54], [55], NUMT20 in its unique motif and NUMT2 in either or both motifs. A red star shows a significant difference between the mutated sequence and the corresponding original sequence (n = 6; p<0.05, Mann-Whitney test). In vector pIRT2Δ, mutation of the key ACS motif in ARS1 and in ARS121 increased the plasmid loss rates to match those of plasmids lacking known origins (∼0.070), as expected. This was also the case when mutating the single ACS motif in NUMT20, (≥0.072 in pIRT2Δ). Reduction in replication activity was observed after mutation of one ACS motif (R2) and of both motifs (R2+R1) in NUMT2, while mutation of the single R1 motif was ineffective. However, with the double mutation plasmid loss rate levels were not as high as for the empty plasmid (pIRT2Δ), indicating that additional elements play a role in replication efficiency.
(TIF)

Figure S4 **Plasmid replication measured by dot-colony assay of yeast growth.** Analysis by dot-colony of the replication activity induced by a variety of sequences in the pIRT1Δ vector. For each strain, growth in rich medium (above) and in selective medium (below). Reduction (at generation 12) and essentially arrest (at generation 32) of colony growth in selective media for constructs that lack ARS or NUMTs, or those that carried mutated NUMTs.
(TIF)

Figure S5 **Position of NUMTs at replication profiles on mutants that cause fork stalling.** Representative examples of replication profiles in wild type and mutant strains, data from Crabbé et al. [40]. For each profile the position of the NUMT and of the closest origin (ORI) has been superimposed. Likely ARS indicates the position of non-confirmed origin, according to oriDB.
(TIF)

Figure S6 **Distribution of NUMTs and ARS on the complete replication profile of** ***S. cerevisiae*** **.** The position of NUMTs and of ARS is superimposed on the replication profile of the yeast chromosomes, adapted from reference [42]. The yellow curve represents the replication time; peaks represent origins of replication (early origins are on taller peaks). The position of NUMTs and ARS is shown (green and pink circles, respectively). ARS are named and indicated with a white arrow. NUMTs names are showed in a white rectangle or in a rose rectangle when they are located inside an ARS (NUMTs 13 and 30). The distance between two ARS or between an ARS and a NUMT is indicated in kb. CEN (a violet circle) corresponds to centromere; TEL to telomeric regions; “Early” and “Late” refers to replication, trep to replication time in min.
(TIF)

Figure S7 **Restriction fragment used for to analyse replication of NUMT14 by 2D-gels.** Schematic representation of the DNA restriction fragment used for the analysis of the origin activity of NUMT14 in 2D-gels. In the upper part are indicated the position, the coordinates and the size of the different elements carried by the DNA fragment. In the middle are indicated the position of the likely ARS pro408 using tiled nucleotide arrays NimbleGen [43], the position of the same ARS by genome wide mapping of MCM binding sites [56], and the center of the origin according to ssDNA in HU [44]. The position of the NUMT (not at scale) is also indicated. A bubble arc is detected if DNA replication initiates within the central half of the restriction fragment. The lower part of the scheme shows the size of the left and right arms of DNA fragments centered on four alternative elements, and indicates that NUMT14 but not the likely ARS408, in any of its defined localisations, is centred on the restriction fragment to generate an arc bubble.
(TIF)

Figure S8 **Mutation in NUMTs compared to the mitochondrial sequence.** (**A**) Types of mutations (Pu = purine; Py = pyrimidine; ins = nucleotide insertion; del = nucleotide deletion) in chromosomal NUMTs compared to the mitochondrial sequence. Fifty-six and 48 mutations analysed in ACS and non-ACS motifs, respectively. Mean ± SD. (**B**) Global nucleotide changes in the mutations analysed above in ACS and non-ACS motifs. TA enrich = TA enrichment; GC enrich = GC enrichment, no enrich = no enrichment in either TA or GC content; other nucleotide insertion or deletion. Mean ± SD.
(TIF)

Table S1 **Sequence information of** ***S. cerevisiae*** **NUMTs.**
(TIF)

Table S2 **Sequence information of additional sequences used in this study.**
(TIF)

Table S3 **Replication activity measured by transformation efficiency.**
(TIF)

Table S4 **Distance of NUMTs from replication peaks defined by Raghuraman** ***et al.***
(TIF)

Table S5 **Analysis of the presence of NUMTs in replication origins.**
(TIF)

Table S6 **Identification of additional ACS-like motifs in** ***S. cerevisiae*** **mitochondrial DNA.**
(TIF)

Text S1 **Supporting methods, results and references.**
(DOC)
