# Supplementary figures and images for: Nuclear Mitochondrial DNA Activates Replication in Saccharomyces cerevisiae
Source: PLoS One. 2011 Mar 8;6(3):e17235. doi: 10.1371/journal.pone.0017235 (PMC3050842; doi:10.1371/journal.pone.0017235)

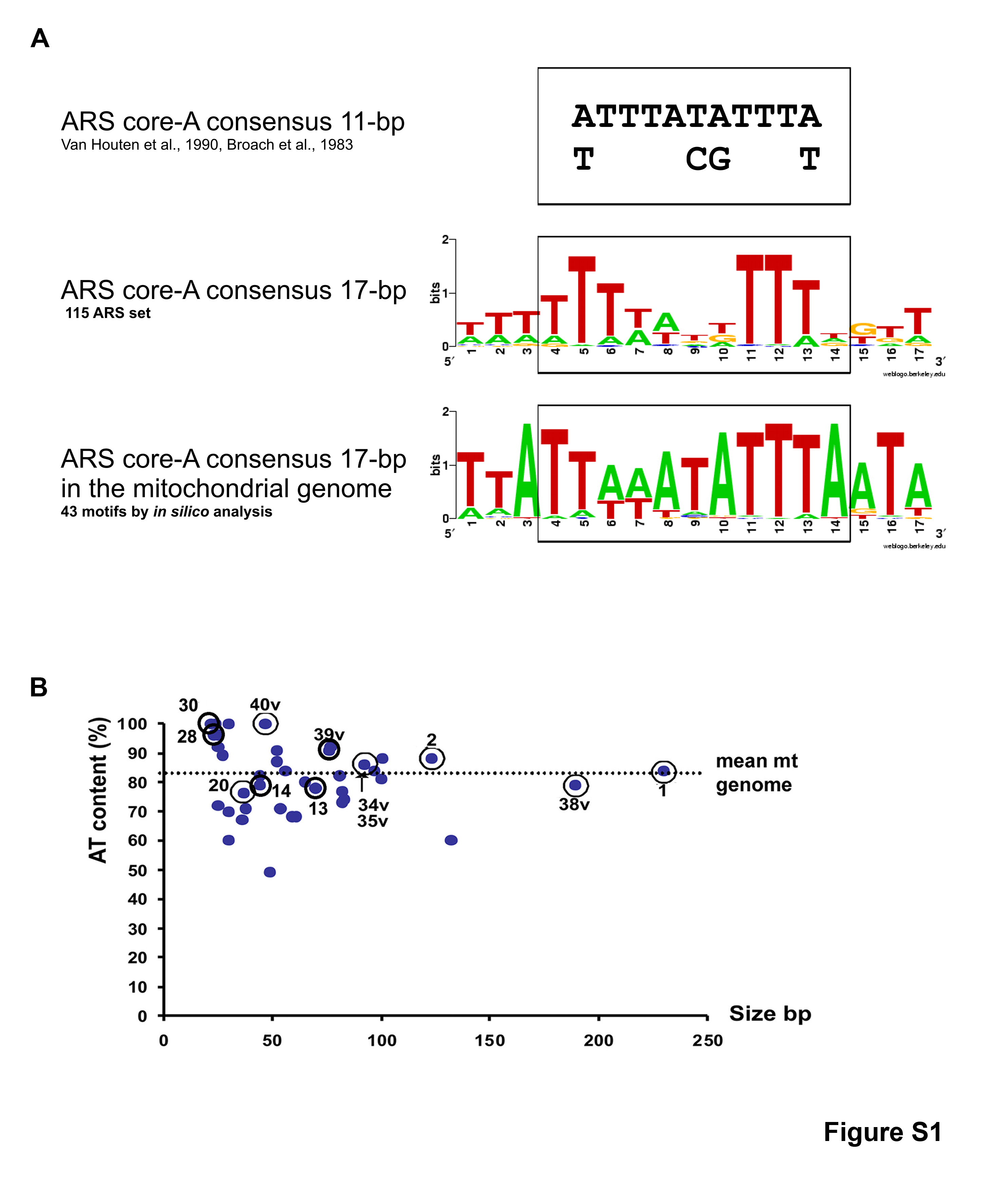

Supplement: Figure S1 — ARS Core-A consensus sequences (ACS). (A) Current 11 bp ARS core-A consensus sequence according to references [30], [51] (top). LOGO representation of the 17 bp-ARS core-A consensus sequence calculated on a set of 115 ARS available in the OriDB database and used in this study (middle). LOGO representation of the 17 bp consensus sequence calculated on a set of 43 mitochondrial ACS motifs (below). Square represents the 11-bp consensus. LOGO representation is designed by WebLogo3: Public Beta [52], [53]. (B) The size of each S. cerevisiae NUMT is plotted against the AT content. Circled NUMTs were investigated in this paper. (TIF) [file pone.0017235.s001.tif]

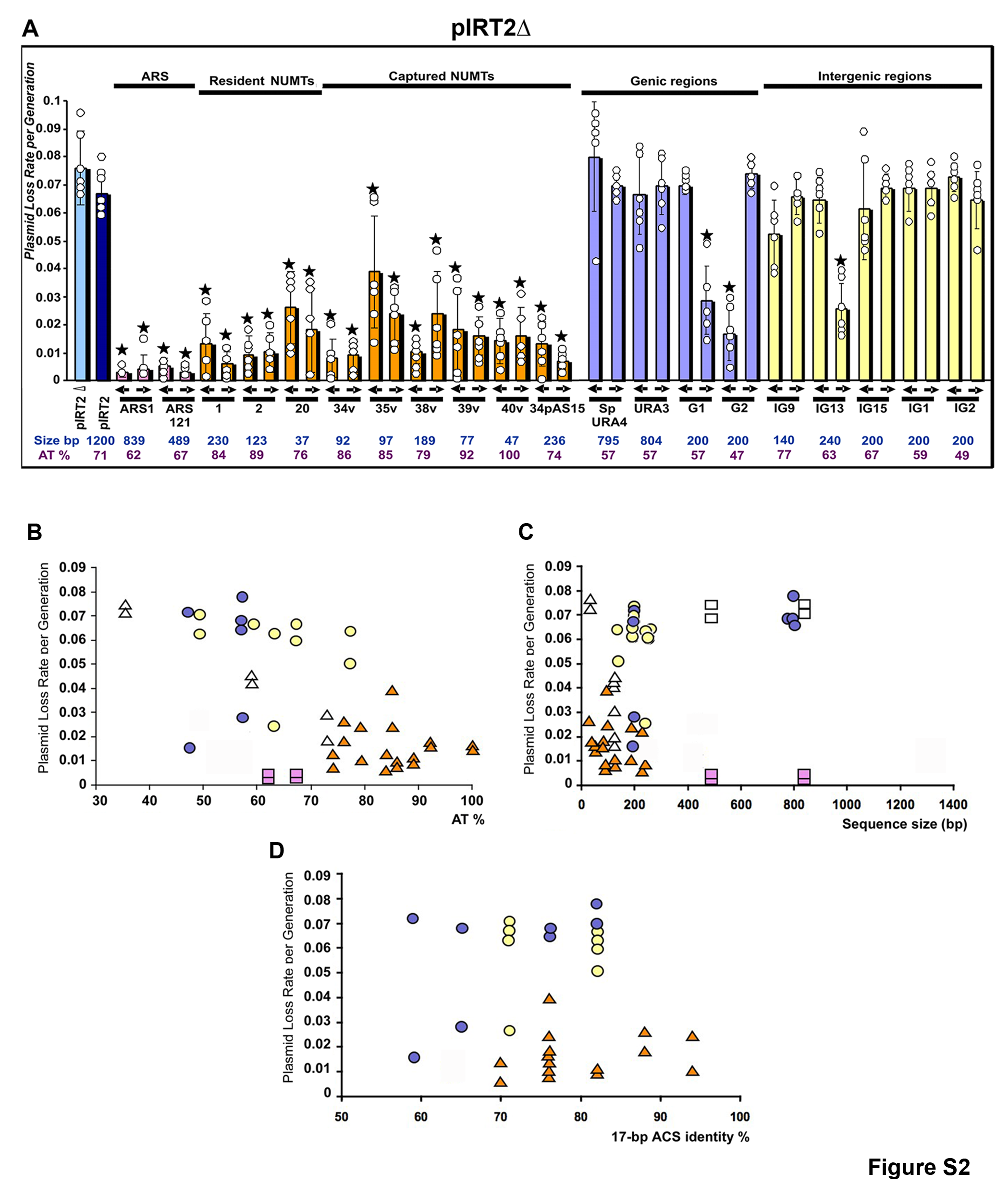

Supplement: Figure S2 — Plasmid loss rates and sequence features analysis of the ectopic vector pIRT2Δ carrying a variety of sequences. Plasmid loss rate per generation measured in 6 independent transformants (circles) per construct (average ± standard deviation). (A) Analysis with vector pIRT2Δ. For each sequence, the insertion was analyzed in both orientations (forward and reverse arrow). When values are identical, only one circle is shown. A black star indicates a difference versus empty pIRT2Δ, according to Mann-Whitney test (p<0.05). pIRT2Δ (no known ARS) and pIRT2 (containing an origin of replication of S. pombe, ARS1 that is expected to be inactive in S. cerevisiae) represent negative controls. ARS1 and ARS121 are positive controls. Plasmid loss rate plotted against (B) the AT content (%), (C) the sequence size, and (D) the identity to the 17 bp-ACS consensus. Framed orange triangles represent S. cerevisiae NUMTs. ARS are indicated with framed purple squares and other sequences with framed circles (yellow for intergenic and blue for genic sequences). Mutated sequences (“m”) are represented by empty triangles (NUMTs) or empty squares (ARS). Original NUMTs and ARS show low plasmid loss rate (high replication) independently from the parameter analysed on the X-axis. Data refer to experiments performed with pIRT2Δ. (TIF) [file pone.0017235.s002.tif]

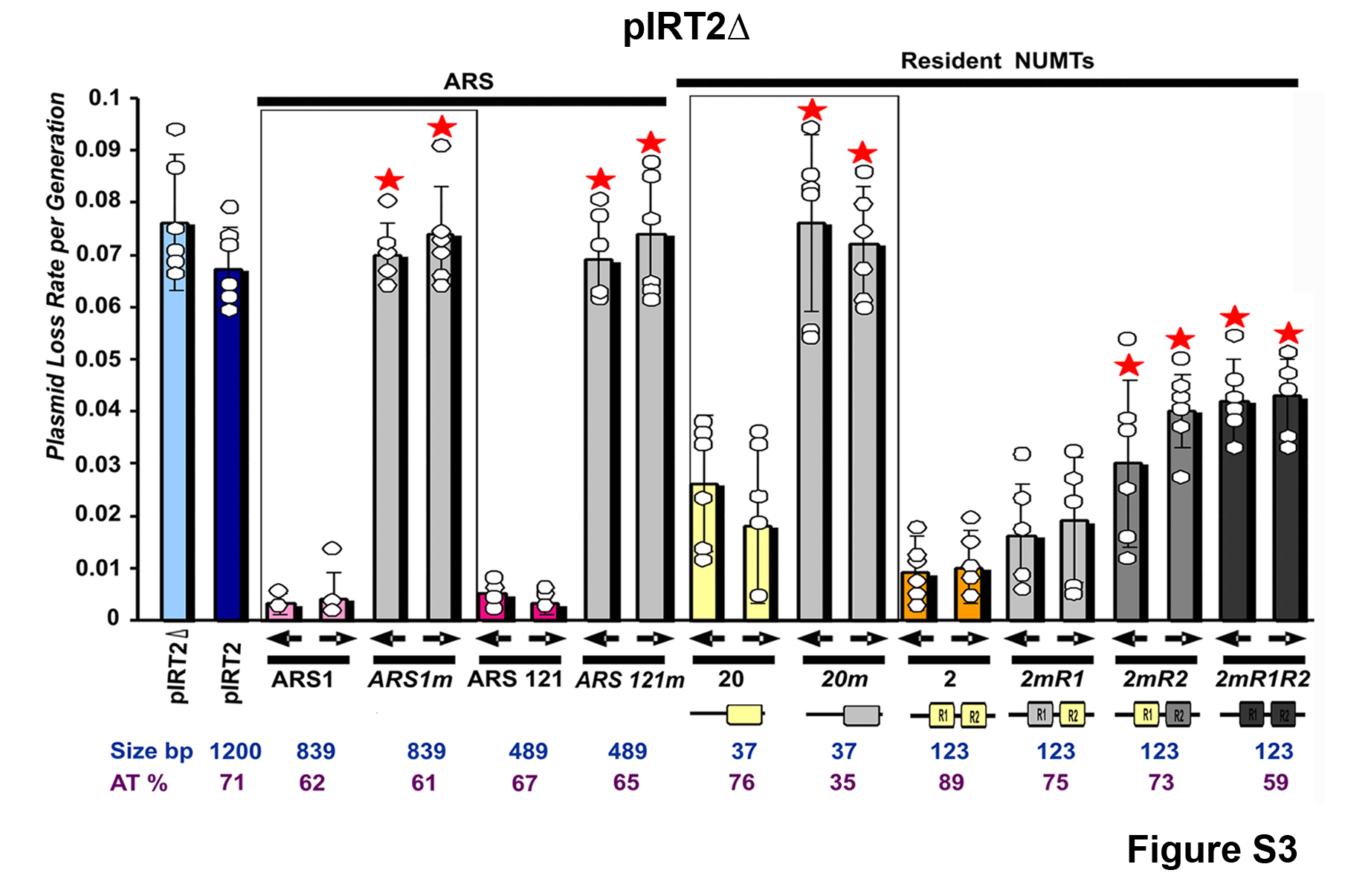

Supplement: Figure S3 — Replication activity with mutated ACS. Plasmid loss rate following ARS and NUMT mutagenesis (“m”) to replace the 17-bp ACS motif in vectors pIRT2Δ. (A) Mutated sequences are represented by grey towers; different intensities of grey are used for mutation of more than one motif. ARS are mutated in the ARS core-A motif [54], [55], NUMT20 in its unique motif and NUMT2 in either or both motifs. A red star shows a significant difference between the mutated sequence and the corresponding original sequence (n = 6; p<0.05, Mann-Whitney test). In vector pIRT2Δ, mutation of the key ACS motif in ARS1 and in ARS121 increased the plasmid loss rates to match those of plasmids lacking known origins (∼0.070), as expected. This was also the case when mutating the single ACS motif in NUMT20, (≥0.072 in pIRT2Δ). Reduction in replication activity was observed after mutation of one ACS motif (R2) and of both motifs (R2+R1) in NUMT2, while mutation of the single R1 motif was ineffective. However, with the double mutation plasmid loss rate levels were not as high as for the empty plasmid (pIRT2Δ), indicating that additional elements play a role in replication efficiency. (TIF) [file pone.0017235.s003.tif]

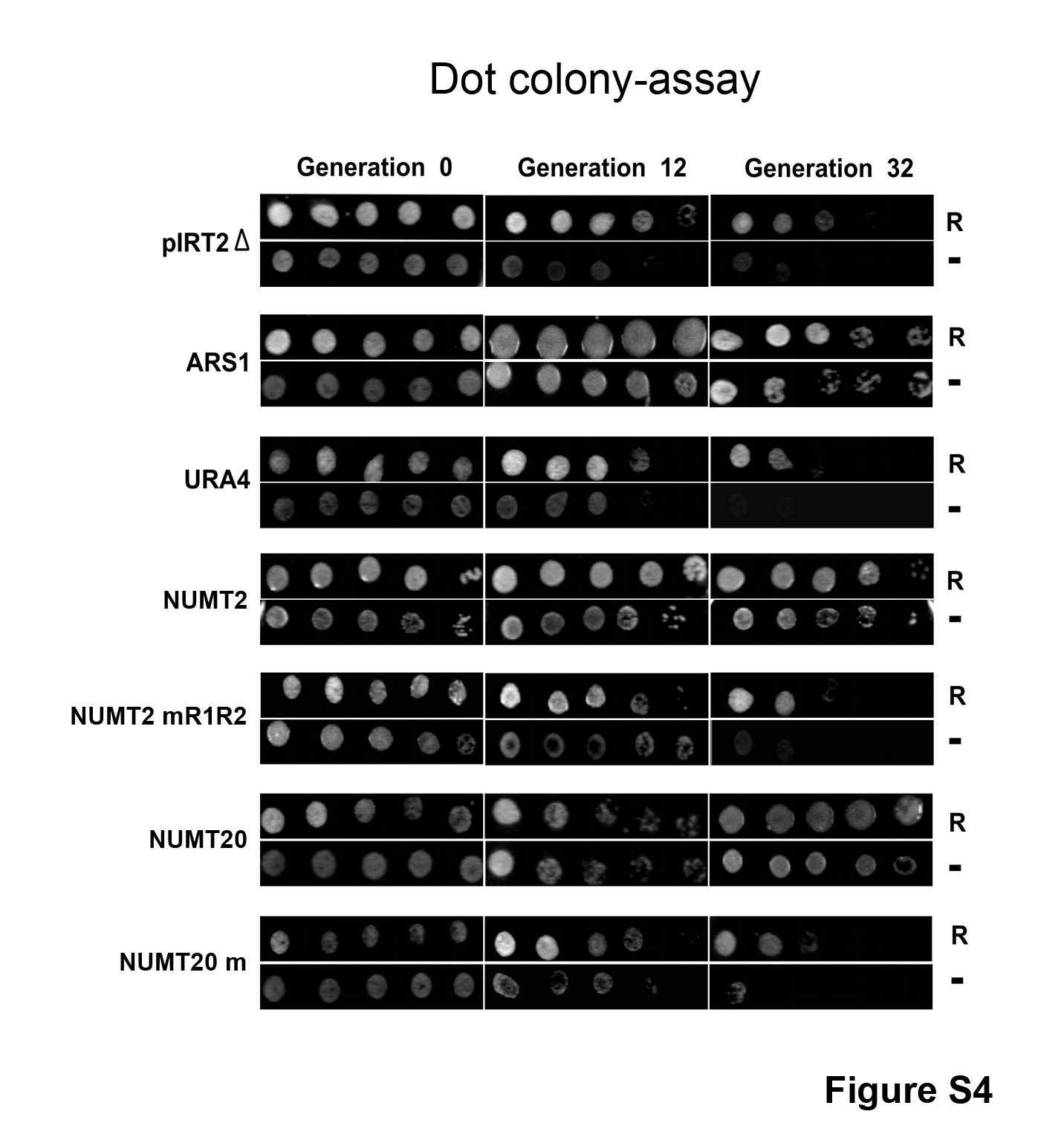

Supplement: Figure S4 — Plasmid replication measured by dot-colony assay of yeast growth. Analysis by dot-colony of the replication activity induced by a variety of sequences in the pIRT1Δ vector. For each strain, growth in rich medium (above) and in selective medium (below). Reduction (at generation 12) and essentially arrest (at generation 32) of colony growth in selective media for constructs that lack ARS or NUMTs, or those that carried mutated NUMTs. (TIF) [file pone.0017235.s004.tif]

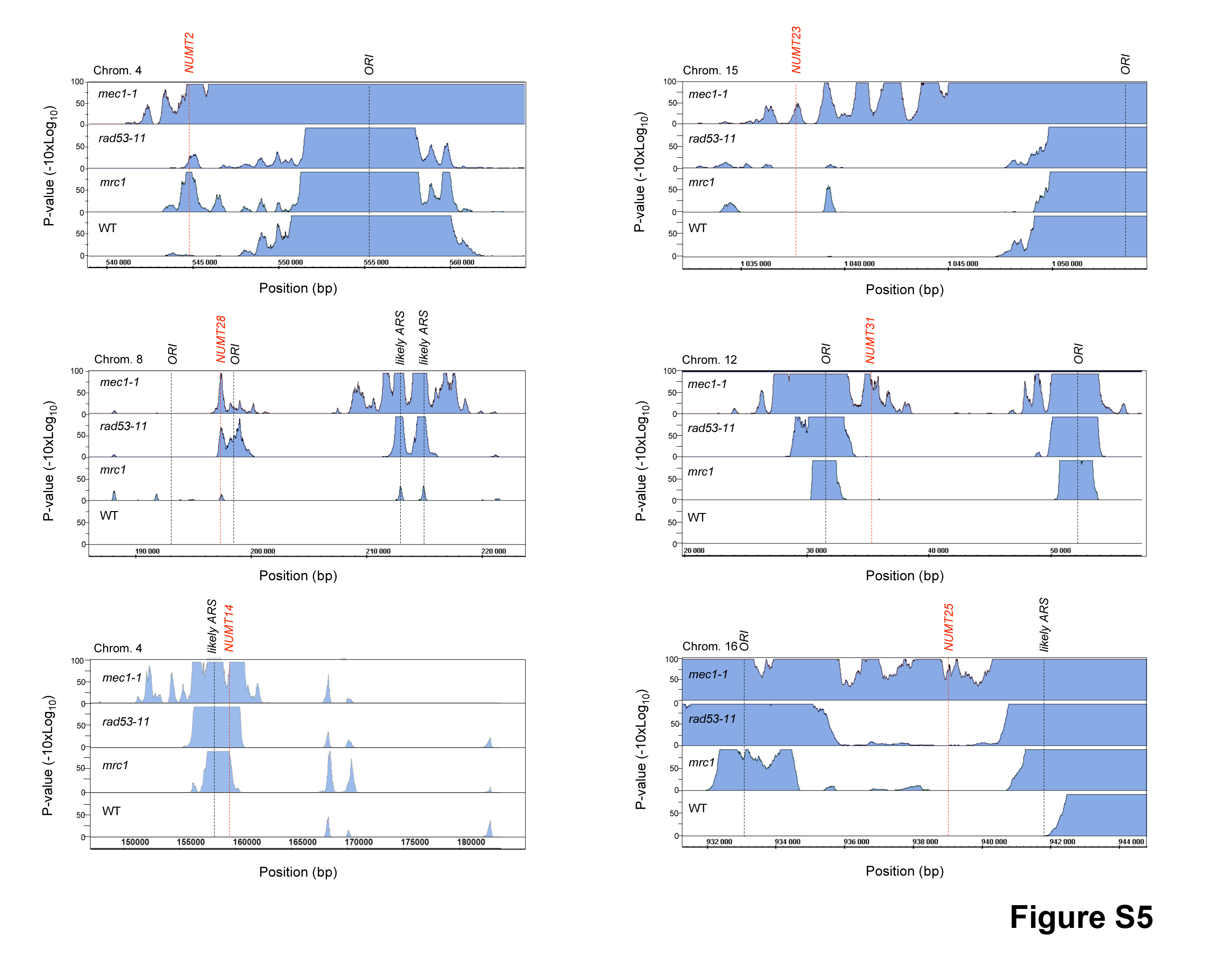

Supplement: Figure S5 — Position of NUMTs at replication profiles on mutants that cause fork stalling. Representative examples of replication profiles in wild type and mutant strains, data from Crabbé et al. [40]. For each profile the position of the NUMT and of the closest origin (ORI) has been superimposed. Likely ARS indicates the position of non-confirmed origin, according to oriDB. (TIF) [file pone.0017235.s005.tif]

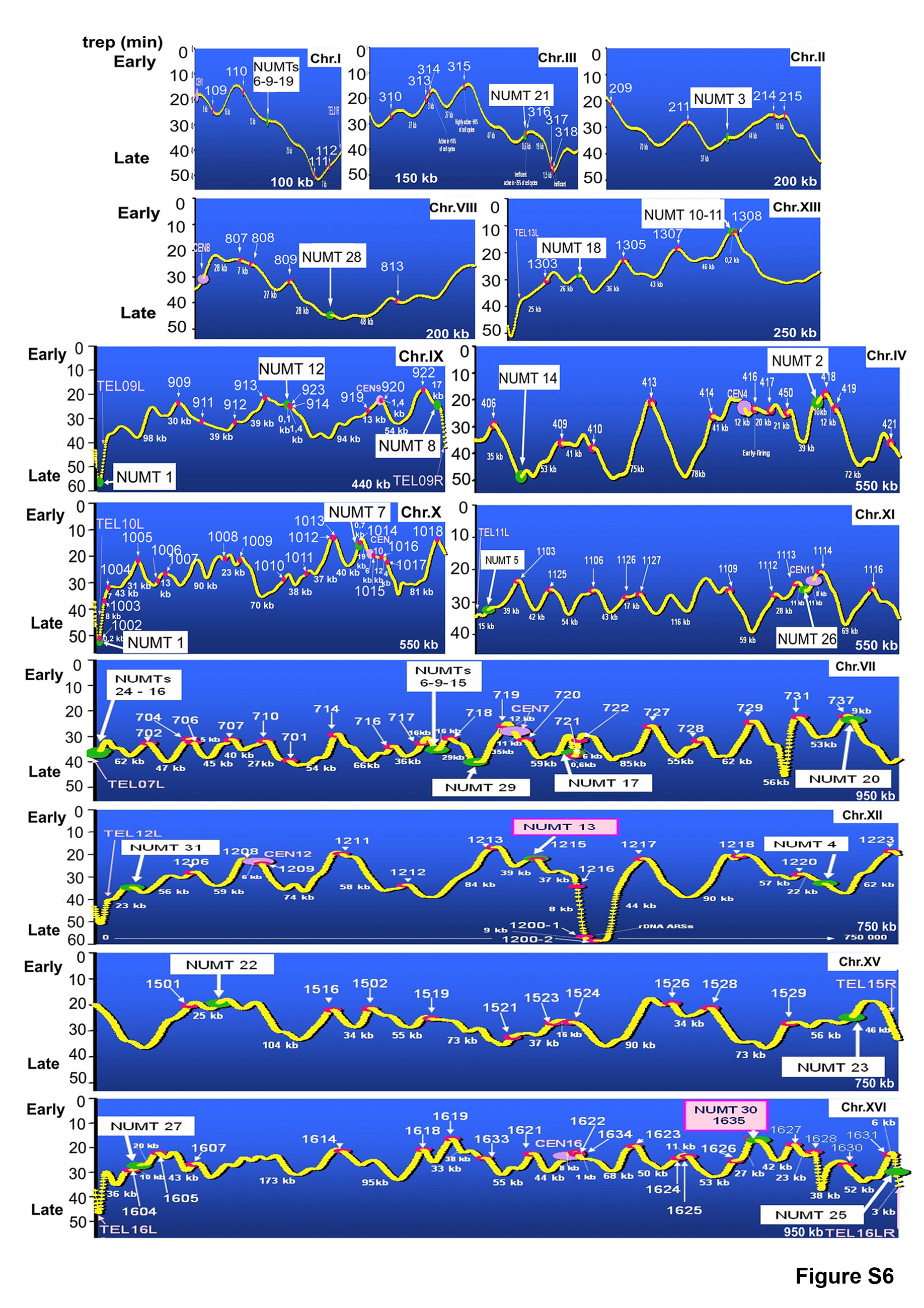

Supplement: Figure S6 — Distribution of NUMTs and ARS on the complete replication profile of S. cerevisiae . The position of NUMTs and of ARS is superimposed on the replication profile of the yeast chromosomes, adapted from reference [42]. The yellow curve represents the replication time; peaks represent origins of replication (early origins are on taller peaks). The position of NUMTs and ARS is shown (green and pink circles, respectively). ARS are named and indicated with a white arrow. NUMTs names are showed in a white rectangle or in a rose rectangle when they are located inside an ARS (NUMTs 13 and 30). The distance between two ARS or between an ARS and a NUMT is indicated in kb. CEN (a violet circle) corresponds to centromere; TEL to telomeric regions; “Early” and “Late” refers to replication, trep to replication time in min. (TIF) [file pone.0017235.s006.tif]

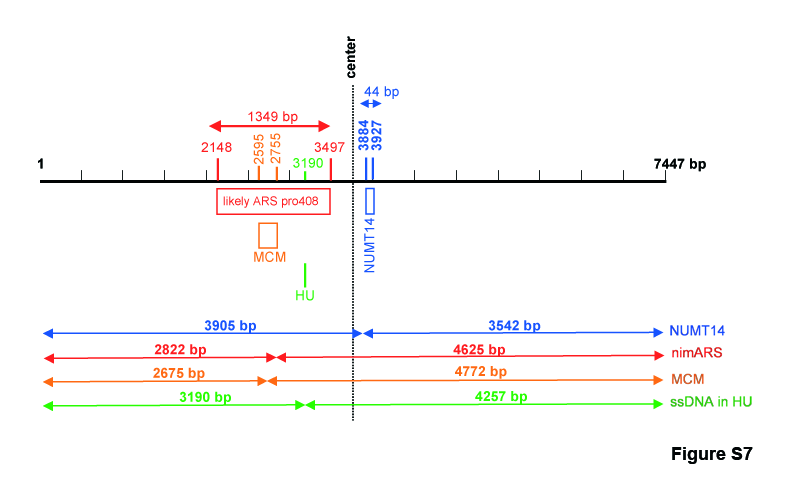

Supplement: Figure S7 — Restriction fragment used for to analyse replication of NUMT14 by 2D-gels. Schematic representation of the DNA restriction fragment used for the analysis of the origin activity of NUMT14 in 2D-gels. In the upper part are indicated the position, the coordinates and the size of the different elements carried by the DNA fragment. In the middle are indicated the position of the likely ARS pro408 using tiled nucleotide arrays NimbleGen [43], the position of the same ARS by genome wide mapping of MCM binding sites [56], and the center of the origin according to ssDNA in HU [44]. The position of the NUMT (not at scale) is also indicated. A bubble arc is detected if DNA replication initiates within the central half of the restriction fragment. The lower part of the scheme shows the size of the left and right arms of DNA fragments centered on four alternative elements, and indicates that NUMT14 but not the likely ARS408, in any of its defined localisations, is centred on the restriction fragment to generate an arc bubble. (TIF) [file pone.0017235.s007.tif]

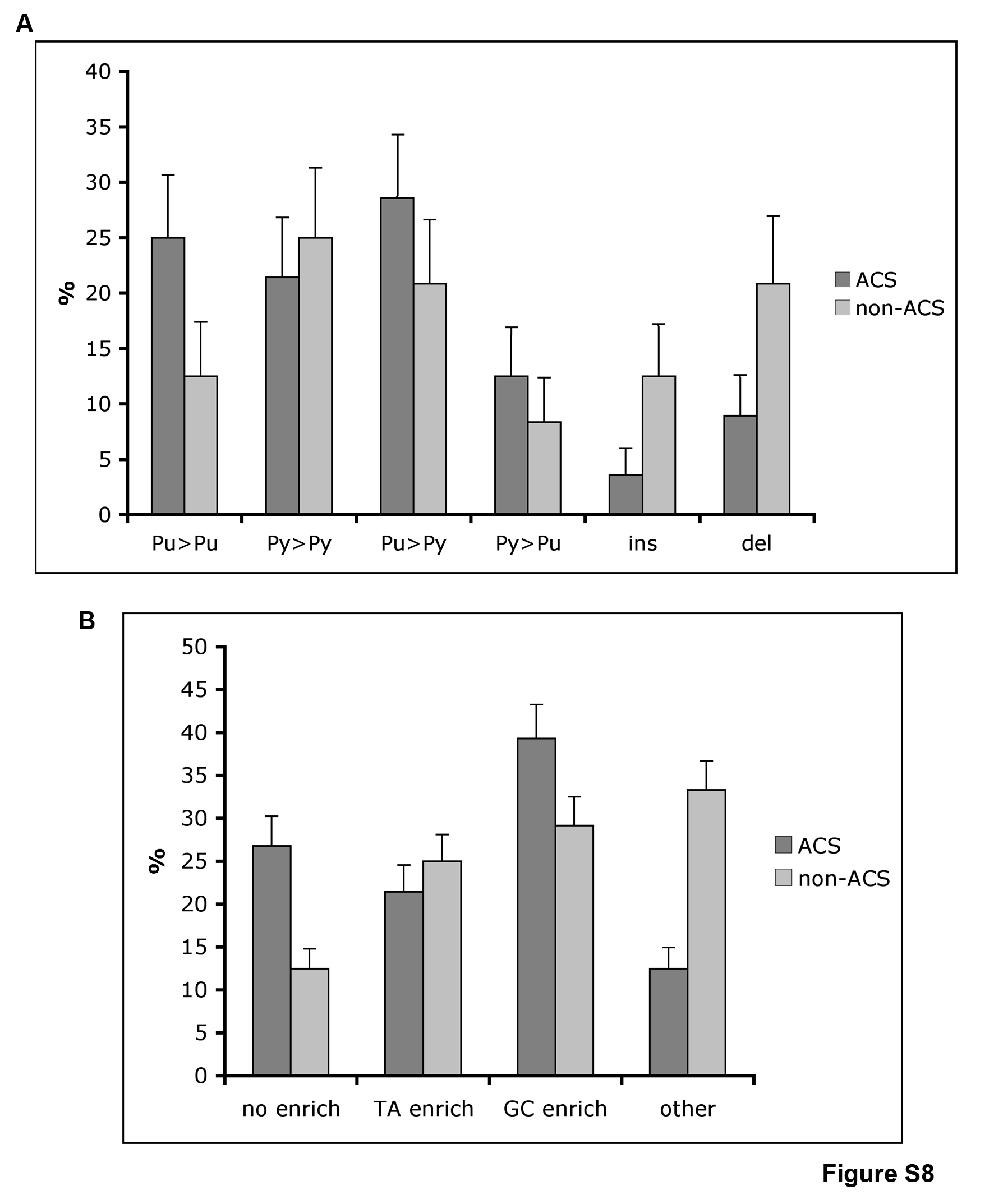

Supplement: Figure S8 — Mutation in NUMTs compared to the mitochondrial sequence. (A) Types of mutations (Pu = purine; Py = pyrimidine; ins = nucleotide insertion; del = nucleotide deletion) in chromosomal NUMTs compared to the mitochondrial sequence. Fifty-six and 48 mutations analysed in ACS and non-ACS motifs, respectively. Mean ± SD. (B) Global nucleotide changes in the mutations analysed above in ACS and non-ACS motifs. TA enrich = TA enrichment; GC enrich = GC enrichment, no enrich = no enrichment in either TA or GC content; other nucleotide insertion or deletion. Mean ± SD. (TIF) [file pone.0017235.s008.tif]

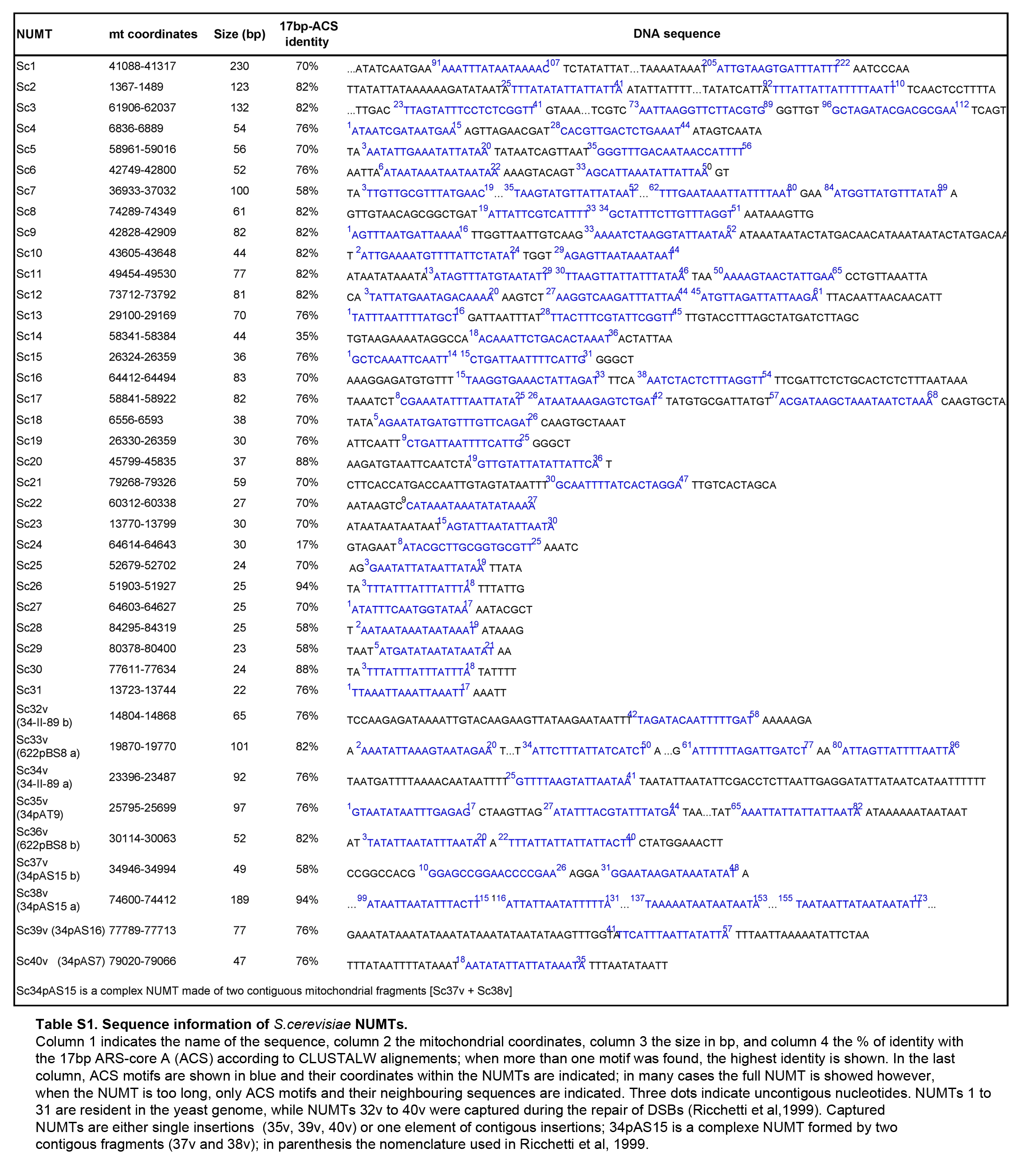

Supplement: Table S1 — Sequence information of S. cerevisiae NUMTs. (TIF) [file pone.0017235.s009.tif]

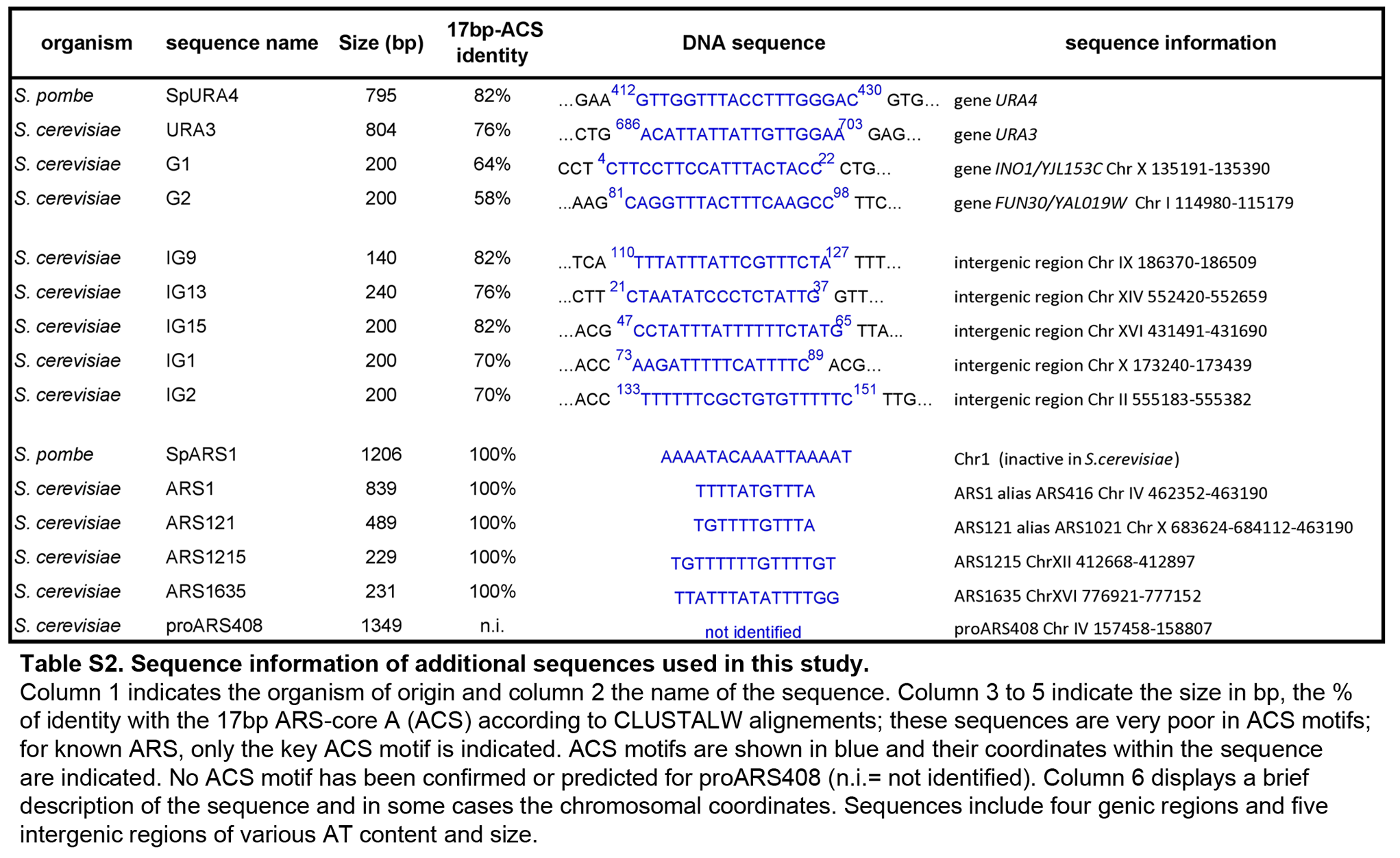

Supplement: Table S2 — Sequence information of additional sequences used in this study. (TIF) [file pone.0017235.s010.tif]

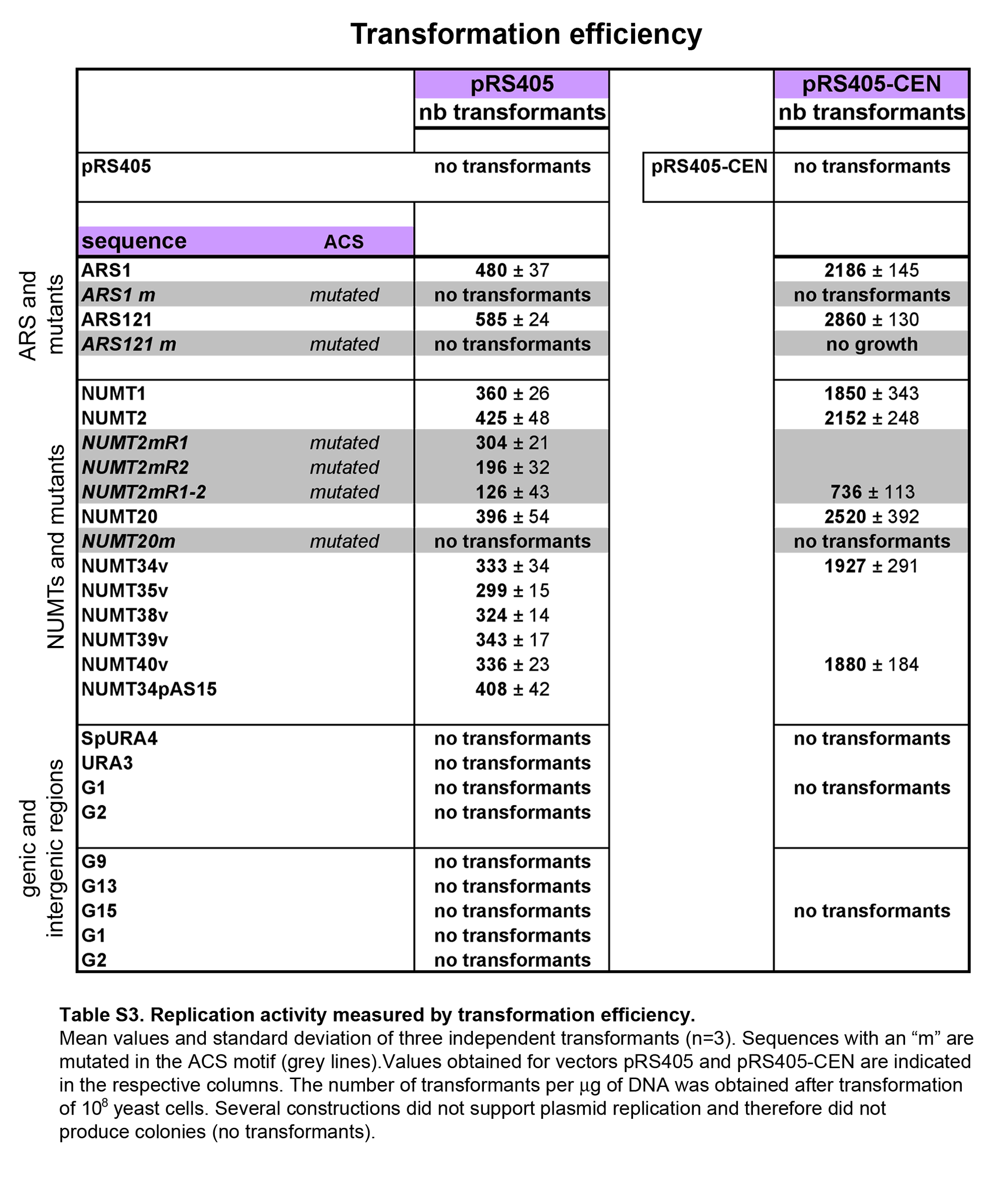

Supplement: Table S3 — Replication activity measured by transformation efficiency. (TIF) [file pone.0017235.s011.tif]

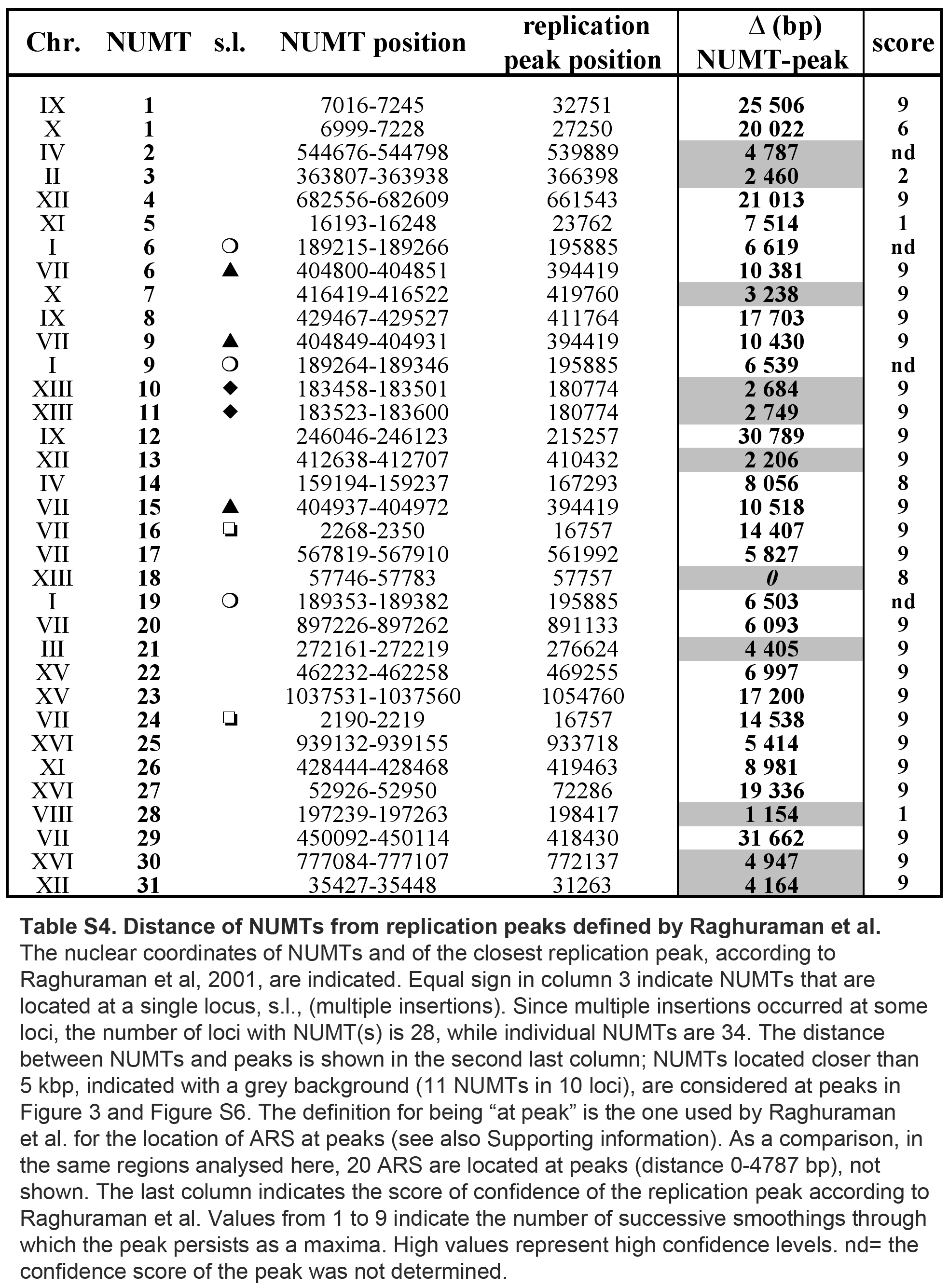

Supplement: Table S4 — Distance of NUMTs from replication peaks defined by Raghuraman et al. (TIF) [file pone.0017235.s012.tif]

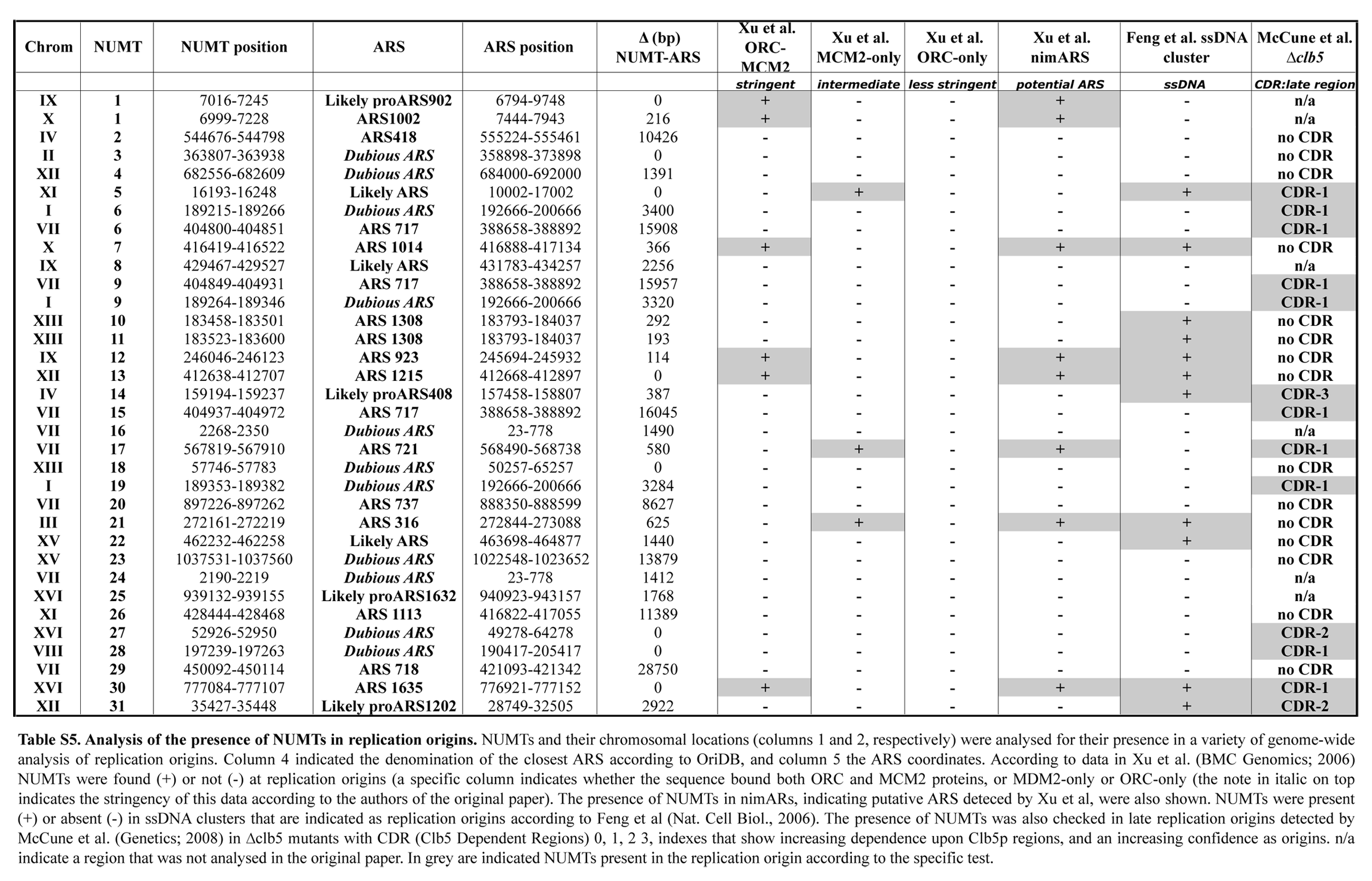

Supplement: Table S5 — Analysis of the presence of NUMTs in replication origins. (TIF) [file pone.0017235.s013.tif]

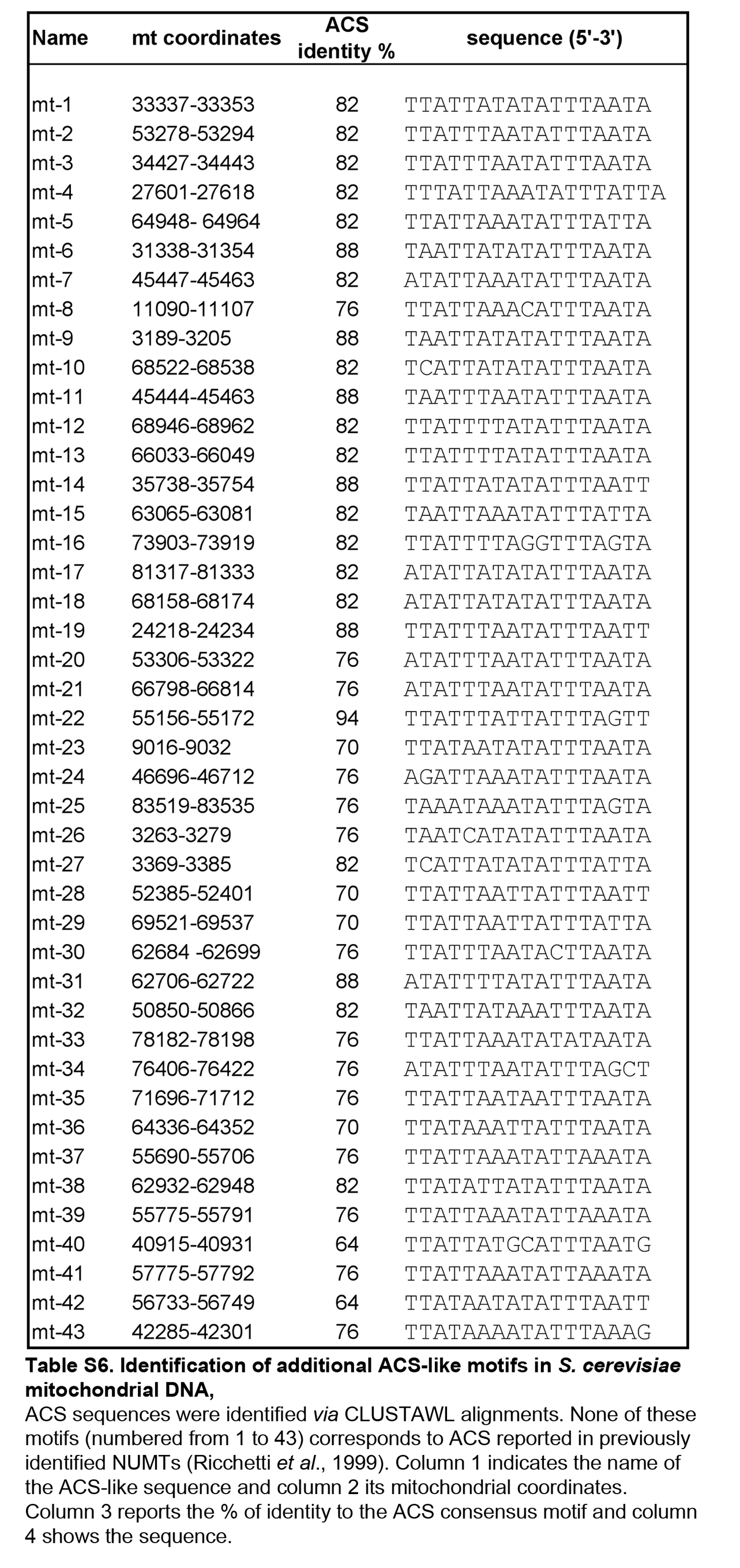

Supplement: Table S6 — Identification of additional ACS-like motifs in S. cerevisiae mitochondrial DNA. (TIF) [file pone.0017235.s014.tif]
