# Supplementary material for: Nuclear Mitochondrial DNA Activates Replication in Saccharomyces cerevisiae
Source: PLoS One. 2011 Mar 8;6(3):e17235. doi: 10.1371/journal.pone.0017235 (PMC3050842; doi:10.1371/journal.pone.0017235)
Supplement: Text S1 — Supporting methods, results and references. (DOC) [file pone.0017235.s015.doc]

# Supporting Text S1

**Method**

**Plasmid replication assay (Dot colony assay).** Single yeast colonies harboring plasmids were resuspended or diluted to an OD_600_ of 0.1, and grown in rich, nonselective medium for 12 or 32 generations (for a period of 18 h or 48 h, respectively) at 30°C. The starting point of growth in nonselective medium was taken at generation zero (G0). Each culture from G0, G12, and G32 was diluted to an OD_600_ of 0.1 and further diluted by 10-fold serial dilutions. For each sample, 5 µL of each dilution were spotted on either rich or selective agar medium for a qualitative analysis of the plasmid retention [1].

**Results**

**Plasmid loss rate of NUMT, ARS and control sequences inserted in pIRT2∆**

Plasmid loss rates were measured in pIRT2∆, a *S. pombe* plasmid that replicates very poorly in *S. cerevisiae*. Significantly, in pIRT2∆ all plasmids containing an ARS or a NUMT showed low plasmid loss rates (0.003-0.005 and 0.007-0.039, respectively) and consequently high replication activity, compared to controls that lacked robust origins (0.076) (Figure S2A). Resident NUMTs were only single insertions, while captured NUMTs included single insertions (34v), contiguous insertions of two unrelated mitochondrial fragments (34pAS15, constituted by NUMTs 37v and 38v), and one element of a contiguous insertion (38v, and 40v) [2]. Interestingly, NUMT38v alone and the full 34pAS15 (37v + 38v) had a similar plasmid loss rates indicating that two joined mitochondrial sequences, captured at a DSB [2], were no more efficient as a replicating entity than one sequence alone, at least as an episome. In conclusion, all ARS and NUMTs were efficient in both orientations, although NUMTs were remarkably shorter (37-236 bp) than ARS (489-839 bp). These data indicate that the insertion of NUMTs enhances dramatically the replication activity of poorly replicating vectors as confirmed nuclear origins do.

**Absence of correlation between plasmid loss rates and AT-richness of the sequence**

The plasmid loss rates were plotted against 1) size of the insert; 2) the level of identity to the ACS motif; or 3) the AT richness of the sequence, and in none of the three cases a correlation was found (Figure S2 B-D). However, we note that some of the most efficient NUMTs were >85% AT-rich. Control plasmids, containing random genic and intergenic regions of the same size or AT content as the NUMTs, generally showed plasmid loss rates as high as controls that lack origins; there were a few exceptions that were active only in one orientation with pIRT2∆, and not with the pRS405 and pRS405-CEN vectors (see Figure S2A and Figure 2A,B). Therefore we cannot ascribe our findings to random AT-richness of the sequences. Conversely, some random control sequences that also contain ACS motifs (identity to the consensus ≥ 67%) had a high plasmid loss rates (≥ 0.051).

**Dot colony assay**

Dot colony assay [1] of yeast grown in rich and selective media shows a reduction (at generation 12) and essentially an arrest (at generation 32) of colony growth in selective media for pIRT2∆ constructs that lack ARS or NUMTs, or those that carried mutated NUMTs (Figure S4), in agreement with plasmid loss rate, transformation efficiency and *Dpn*I resistance assays.

**Positioning of NUMTs on additional replication profiles**

The distance between NUMTs and peaks (as by Raghuraman, 2001) is scaled in Figure 3 and in Figure S6. We used a distance of 5 kbp as cut off for association (this value was chosen as in Raghuraman’s paper known origins fitted with replication peaks profiles within a distance ≤5 kbp; a mean distance of 3.7 kbp between the predicted origins and centers of known origin windows was found), Table S4. With this cut-off, we found that 10 out of 28 NUMTs were located at peaks. As comparison, using the same cut-off, 20 out of 33 ARS analysed in the same chromosome regions, are correlated to peaks (not shown in Table S4).

**Non-coincidental location of NUMTs close to ARS**

Table 1 shows that 16/34 (47%) of resident NUMTs are located within 650 bp from an ARS. Nine out of these 16 ARS are “confirmed”, 3 are “likely”, and 4 are “dubious” ARS, according to oriDB (<http://www.oridb.org/index.php>). Confirmed origins indicate ARS that have been cloned and detected by ARS assay (support of plasmid replication) or detected by 2D-gels. Likely ARS have been identified by two or more microarray studies, and dubious ARS are detected as origins by only one microarray-based study.

To check whether the high number of NUMTs close to ARS is significant, we compared the actual and the expected distribution of NUMT close to ARS. The average size of confirmed ARS is 440 bp (calculated on 325 ARS), of likely ARS is 2,575 bp (calculated on 225 ARS), and of dubious ARS is 7,033 (calculated on 164 ARS). The portion of the genome occupied by ARS was calculated taking into account the number of confirmed, likely and dubious ARS and their average size, plus a 650 bp sequence on each side of the ARS, which represents all together 22.8% of the yeast nuclear genome (2.85x10^6^ bp out of 1.25x10^7^ bp). The expected value is thus significantly lower than the observed value, 47%, (alpha < 0.001, chi-squared test). The presence of NUMTs close to ARS is even more significant when we take into account only NUMTs located close to confirmed ARS (9/34 or 26.5%), while confirmed ARS and 650 bp sequence on each side represent 4.5% of the total nuclear genome (alpha < 0.001, chi-squared test). These data indicate that the insertion of NUMTs close to ARS is not coincidental, and that this is particularly the case for NUMTs located close to confirmed origins.

**Identification of additional ACS sequences in the mitochondrial genome**

To investigate which fraction of the ACS motifs present in the mitochondrial genome corresponds to fragments integrated in the nucleus, we uded CLUSTALW alignments of the complete yeast mitochondrial genome (85.8 kbp in size) [3]. We found that, in addition to regions corresponding to resident or captured NUMTs, at least 43 other yeast mitochondrial regions display ACS identity (64-94%) (Table S6). This indicates that the mitochondrial DNA contains more ACS motifs than those already transferred to the nuclear genome, and suggests that new insertions may also contain ACS motifs.

These 43 ACS motifs, each 17 bp long, added to 53 and 19 ACS motifs present in resident and captured NUMTs, respectively (indicated in Table S1), represent 1/44 of the total mitochondrial genome (1955 bp/85779 bp). Although this is not an exclusive list and certainly other ACS motifs with a lower identity to known ACS can be added, we consider that it is reasonably indicative of the proportion of the mitochondrial genome occupied by ACS. In conclusion the mitochondrial genome by itself is not necessarily ACS-rich.

**Mutations of NUMTs in the nuclear genome**

We previously showed that the sequence of most resident NUMTs is not identical to that of the mitochondrial DNA [2], compatibly with mutations accumulated after integration in the nuclear genome. Accordingly, captured NUMTs that are recent insertions are 100% identical to sequences of the mitochondrial DNA. By sequence analysis we investigated whether mutations that occurred after the integration of NUMTs in the nuclear genome may affect the replication activity of these sequences. For simplicity we did not take into account mutations that may have occurred in the mitochondrial genome since the insertion occurred. We found that the mutations were not significantly different in percentage in ACS versus non-ACS motifs (6.97% versus 4.27%, respectively, p= 0.17). Moreover, out of 104 mutations detected, we observed global pyrimidine enrichment, since purine versus pyrimidine changes were more frequent that pyrimidine versus purine changes, both in ACS and non-ACS sequences (Figure S8A). Furthermore we observed a prevalent GC than AT enrichment, again both in ACS and non-ACS sequences (Figure S8B). However, nucleotide insertions and deletions were more frequent in non-ACS than in ACS motifs. We observed that resident NUMTs that promoted replication in plasmid loss rates tests (NUMTS 1, 2 and 20) had no mutations in the ACS motifs. Moreover, NUMTs that showed replication activity also on chromosomes (NUMTs 14, and 28) had mutations in the ACS motifs that either decreased or increased the ACS consensus by one base, respectively (not shown). Thus, by sequence analysis, mutations in ACS motifs of NUMTs do not appear either to promote or inhibit replication, at least with the present set of data.

**References**

1. Chattopadhyay A, Schmidt MC, Khan SA (2005) Identification of a 450-bp region of human papillomavirus type 1 that promotes episomal replication in Saccharomyces cerevisiae. Virology 340: 133-142.

2. Ricchetti M, Fairhead C, Dujon B (1999) Mitochondrial DNA repairs double-strand breaks in yeast chromosomes. Nature 402: 96-100.

3. Foury F, Roganti T, Lecrenier N, Purnelle B (1998) The complete sequence of the mitochondrial genome of Saccharomyces cerevisiae. FEBS Lett 440: 325-331.

4. Broach JR, Li YY, Feldman J, Jayaram M, Abraham J, et al. (1983) Localization and sequence analysis of yeast origins of DNA replication. Cold Spring Harb Symp Quant Biol 47 Pt 2: 1165-1173.

5. Van Houten JV, Newlon CS (1990) Mutational analysis of the consensus sequence of a replication origin from yeast chromosome III. Mol Cell Biol 10: 3917-3925.

6. Crooks GE, Hon G, Chandonia JM, Brenner SE (2004) WebLogo: a sequence logo generator. Genome Res 14: 1188-1190.

7. Schneider TD, Stephens RM (1990) Sequence logos: a new way to display consensus sequences. Nucleic Acids Res 18: 6097-6100.

8. Marahrens Y, Stillman B (1992) A yeast chromosomal origin of DNA replication defined by multiple functional elements. Science 255: 817-823.

9. Walker SS, Malik AK, Eisenberg S (1991) Analysis of the interactions of functional domains of a nuclear origin of replication from Saccharomyces cerevisiae. Nucleic Acids Res 19: 6255-6262.

10. Crabbe L, Thomas A, Pantesco V, De Vos J, Pasero P, et al. Analysis of replication profiles reveals key role of RFC-Ctf18 in yeast replication stress response. Nat Struct Mol Biol 17: 1391-1397.

11. Raghuraman MK, Winzeler EA, Collingwood D, Hunt S, Wodicka L, et al. (2001) Replication dynamics of the yeast genome. Science 294: 115-121.

12. Xu W, Aparicio JG, Aparicio OM, Tavare S (2006) Genome-wide mapping of ORC and Mcm2p binding sites on tiling arrays and identification of essential ARS consensus sequences in S. cerevisiae. BMC Genomics 7: 276.

13. Wyrick JJ, Aparicio JG, Chen T, Barnett JD, Jennings EG, et al. (2001) Genome-wide distribution of ORC and MCM proteins in S. cerevisiae: high-resolution mapping of replication origins. Science 294: 2357-2360.

14. Feng W, Collingwood D, Boeck ME, Fox LA, Alvino GM, et al. (2006) Genomic mapping of single-stranded DNA in hydroxyurea-challenged yeasts identifies origins of replication. Nat Cell Biol 8: 148-155.
